# Scalable and high-throughput production of an injectable platelet-rich plasma (PRP)/cell-laden microcarrier/hydrogel composite system for hair follicle tissue engineering

**DOI:** 10.1186/s12951-022-01671-8

**Published:** 2022-11-03

**Authors:** Yufan Zhang, Panjing Yin, Junfei Huang, Lunan Yang, Zhen Liu, Danlan Fu, Zhiqi Hu, Wenhua Huang, Yong Miao

**Affiliations:** 1 Department of Plastic and Aesthetic Surgery, Nanfang Hospital of Southern Medical University, 510515 Guangzhou, Guangdong Province China; 2 Department of Joint Surgery, The Third Affiliated Hospital of Southern Medical University, Southern Medical University, Guangzhou, China; 3 Guangdong Engineering Research Center for Translation of Medical 3D Printing Application, Guangdong Provincial Key Laboratory of Medical Biomechanics, Department of Human Anatomy, School of Basic Medical Sciences, Southern Medical University, Guangzhou, China; 4 Biomaterials Research Center, School of Biomedical Engineering, Southern Medical University, 510515 Guangzhou, PR China

**Keywords:** Dermal papilla cells, Hair follicles, Microcarriers, Platelet-rich plasma, Tissue engineering

## Abstract

**Background:**

Tissue engineering of hair follicles (HFs) has enormous potential for hair loss treatment. However, certain challenges remain, including weakening of the dermal papilla cell (DPC) viability, proliferation, and HF inducibility, as well as the associated inefficient and tedious preparation process required to generate extracellular matrix (ECM)-mimicking substrates for biomolecules or cells. Herein, we utilized gelatin methacryloyl (GelMA) and chitosan hydrogels to prepare scalable, monodispersed, and diameter-controllable interpenetrating network GelMA/chitosan-microcarriers (IGMs) loaded with platelet-rich plasma (PRP) and seeded with DPCs, on a high-throughput microfluidic chip.

**Results:**

The ECM-mimicking hydrogels used for IGMs exhibited surface nano-topography and high porosity. Mass production of IGMs with distinct and precise diameters was achieved by adjusting the oil and aqueous phase flow rate ratio. Moreover, IGMs exhibited appropriate swelling and sustained growth factor release to facilitate a relatively long hair growth phase. DPCs seeded on PRP-loaded IGMs exhibited good viability (> 90%), adhesion, spreading, and proliferative properties (1.2-fold greater than control group). Importantly, PRP-loaded IGMs presented a higher hair inducibility of DPCs in vitro compared to the control and IGMs group (p < 0.05). Furthermore, DPC/PRP-laden IGMs were effectively mixed with epidermal cell (EPC)-laden GelMA to form a PRP-loaded DPC/EPC co-cultured hydrogel system (DECHS), which was subcutaneously injected into the hypodermis of nude mice. The PRP-loaded DECHS generated significantly more HFs (~ 35 per site) and novel vessels (~ 12 per site) than the other groups (p < 0.05 for each).

**Conclusion:**

Taken together, these results illustrate that, based on high-throughput microfluidics, we obtained scalable and controllable production of ECM-mimicking IGMs and DECHS, which simulate an effective micro- and macro-environment to promote DPC bioactivity and hair regeneration, thus representing a potential new strategy for HF tissue engineering.

**Supplementary Information:**

The online version contains supplementary material available at 10.1186/s12951-022-01671-8.

## Background

Recently, HF tissue engineering and hair regeneration have become an emerging research focus. Dermal papilla cells (DPCs), which are accessible mesenchymal stem cells (MSCs) within HFs [1, 2], play a critical role in regulating HF growth, morphogenesis, and regeneration [3]. However, following removal from the HF microenvironment, the hair regeneration-inducing capacity of DPCs decreases with time [4]. Consequently, various approaches have been developed to retain these properties. For example, DPCs can be co-cultured with epidermal cells (EPCs) [5], treated with growth factors [6], keratinocyte growth medium [7], or other small molecules [8], and aggregated as DPC spheroids. Among these, DPC spheroids are conventionally applied in HF tissue engineering using hanging-drop cultures [4] or low adhesion surface materials [9] that facilitate close contact between DPCs, thereby promoting cell-cell interactions and increasing inductivity [10]. However, several limitations associated with these approaches have been noted. First, they can cause loss of the extracellular matrix (ECM) [11], resulting in few interactions between DPCs and the ECM. Second, hypoxia can occur in the center of the spheroid [12], which promotes central necrosis, making it difficult for the central cells to exchange gases and metabolites. Third, DPCs do not readily proliferate in aggregates [13].

Importantly, many studies have reported that anchorage-dependent cells (ADCs), such as DPCs, require attachment to a suitable substrate to achieve cell adhesion, spreading, and proliferation. Indeed, ADCs without spreading exhibit poor settlement or commitment and low proliferation accompanied by cell death and thus, exhibit hindered tissue regeneration [13, 14]. Hence, a novel strategy, such as microcarriers (MCs), is required to facilitate ADC attachment for efficient tissue engineering.

MCs have a high specific surface area for cell adhesion and expansion, thereby reducing the requirement for lab space and the associated costs [15, 16]. MCs can also promote the transfer of gases more efficiently [16] while allowing the addition of cytokines, [17]. Thus, MCs are capable of loading seed cells and delivering them to specific sites when injected internally [18]. In fact, the MC culture system reportedly sustains the original tissue phenotype (e.g., chondrogenic properties) [19, 20]. Therefore, the application of MCs may improve current tissue engineering techniques.

Hydrogels are widely used in biomedicine. Gelatin methacryloyl (GelMA) and chitosan are closely associated with the ECM [21]. In 2018, Suo et al. [22] developed an interpenetrating network (IPN) GelMA/chitosan (GC) hydrogel through photo-crosslinking and basification. This hydrogel exhibited improved mechanical properties and bore more loaded cells without requiring the addition of toxic or chemical crosslinkers while retaining the advantages of the two natural biomaterials [22]. Moreover, the IPN structure improves the spreading properties of ADCs [23], while the surface nano-topography of IPN hydrogels alters the interactions between cells and bioactive materials, thereby further influencing cell behavior and fate [24]. In addition to MC fabrication approaches, traditional methods such as bulk emulsification have certain inherent limitations, including the tedious protocols, non-uniform dispersion of MCs, and batch variability [22]. Meanwhile, the droplet microfluidic chip technology offers a high-throughput method for preparing large quantities of monodispersed MCs as their size can be precisely controlled [25]. Other advantages of this technology include lower reagent consumption, low cost, and automation [26]. However, it also has certain limitations, including variable cell cultivation conditions (nutrients, PH, gases) and cell behaviors in different devices, as well as a lack of integrated equipment (automatic analysis and display equipment) [27–29]. Nevertheless, such technology has considerable application value within the life science fields with the potential to become an important toolset for research and development [25].

Platelet-rich plasma (PRP) has been used to treat patients with androgenetic alopecia [30]. PRP is derived from blood and contains high concentrations of platelets, which burst release multiple growth factors, including platelet-derived growth factor, transforming growth factor-β, vascular endothelial growth factor (VEGF), and epidermal growth factor. These growth factors can contribute to hair regeneration and provide a suitable microenvironment for HF tissue engineering [30, 31].

**Fig. 1:**
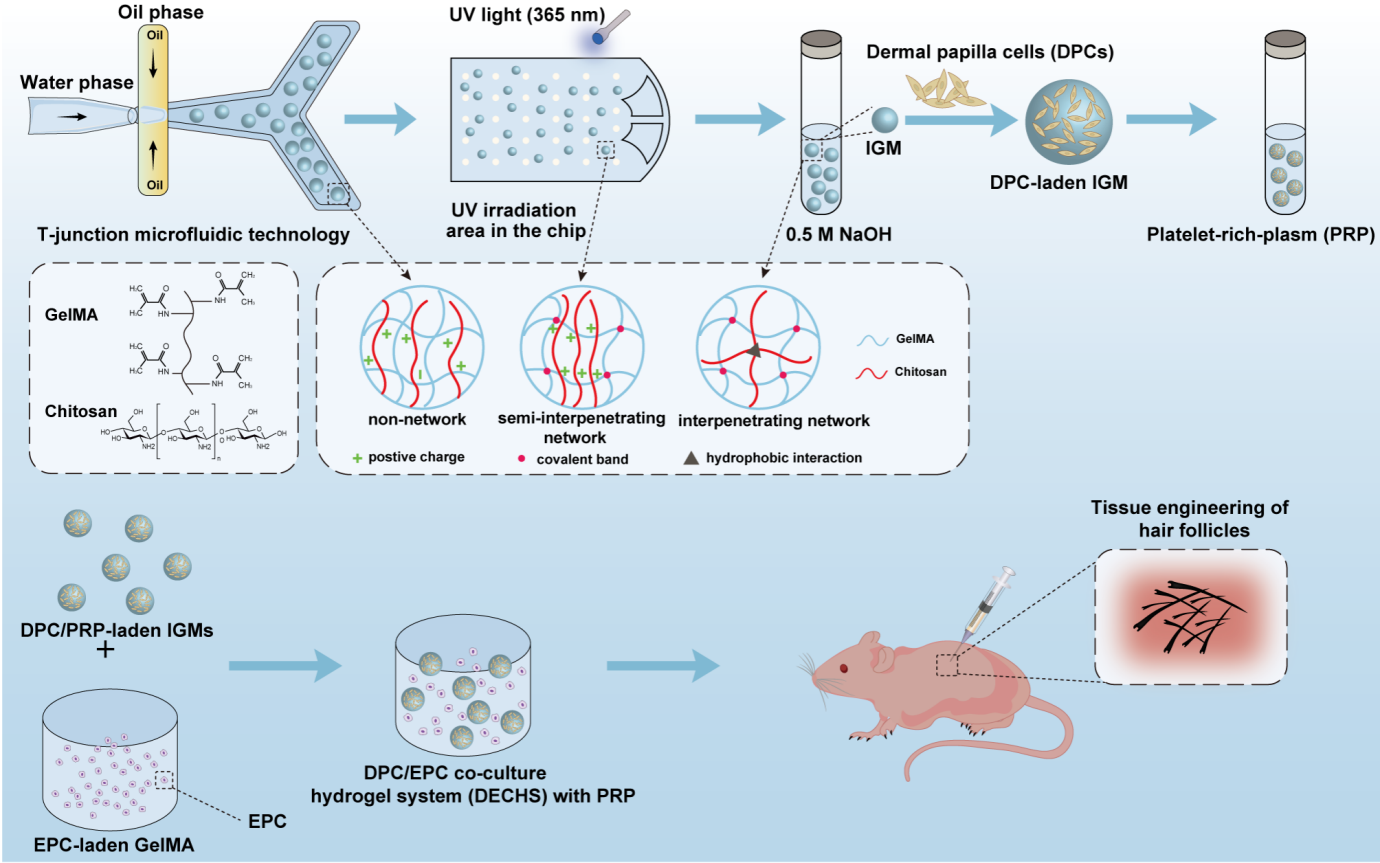
Schematic of the experimental design and preparation process of IPN GC hydrogels

In the current study, we utilized droplet microfluidic technology to prepare large scale and controllable DPC-laden MCs based on GC hydrogels (Fig. 1). We assessed the morphology and properties of the IPN GelMA/chitosan microcarriers (IGMs), as well as the viability, proliferation, and DP signature expression of DPC/PRP-laden IGMs. Finally, DPC/PRP-laden IGMs were mixed with EPC-laden GelMA to form a PRP-loaded DPC/EPC co-cultured hydrogel system (DECHS), which reconstructed interactions between mesenchymal and epithelial components. This system was then injected into nude mice to evaluate its ability to promote hair regeneration. Notably, the DECHS effectively integrates the in vivo HF micro- and macroenvironments; that is, the DPC-laden MCs resemble in vivo DPCs surrounded by an EPC-rich epidermal sheath. Collectively, the findings of this study present a promising approach for HF tissue engineering.

## Results and discussion

### Preparation and characterization of hydrogels

Our team previously reported the design of a surface tension culture to form inductive microtissue [4], and a layer-by-layer culture to reconstruct nanoscale ECM [32–34]. Although we achieved HF induction, the center of the spheroids continued to become hypoxic. Moreover, although pre-vascularized collagen fibers, formed via foreign body reaction [35], and pre-vascularized layer-by-layer microtissues [11] enhance blood perfusion in regenerative tissues, they require complex and relatively inefficient preparation processes. In contrast, a 3D-bioprinted scaffold [36] can better simulate the HF microenvironment, however, is incapable of simulating the associated macroenvironment. Accordingly, to achieve effective hair regeneration, we raised a scalable and high-throughput approach for DPC/PRP-laden microcarriers integrated with EPC-laden hydrogel to mimic the physiological behaviors of DPC, as well as the HF micro- and macroenvironment, while improving the efficiency and output of the preparation process.

**Fig. 2:**
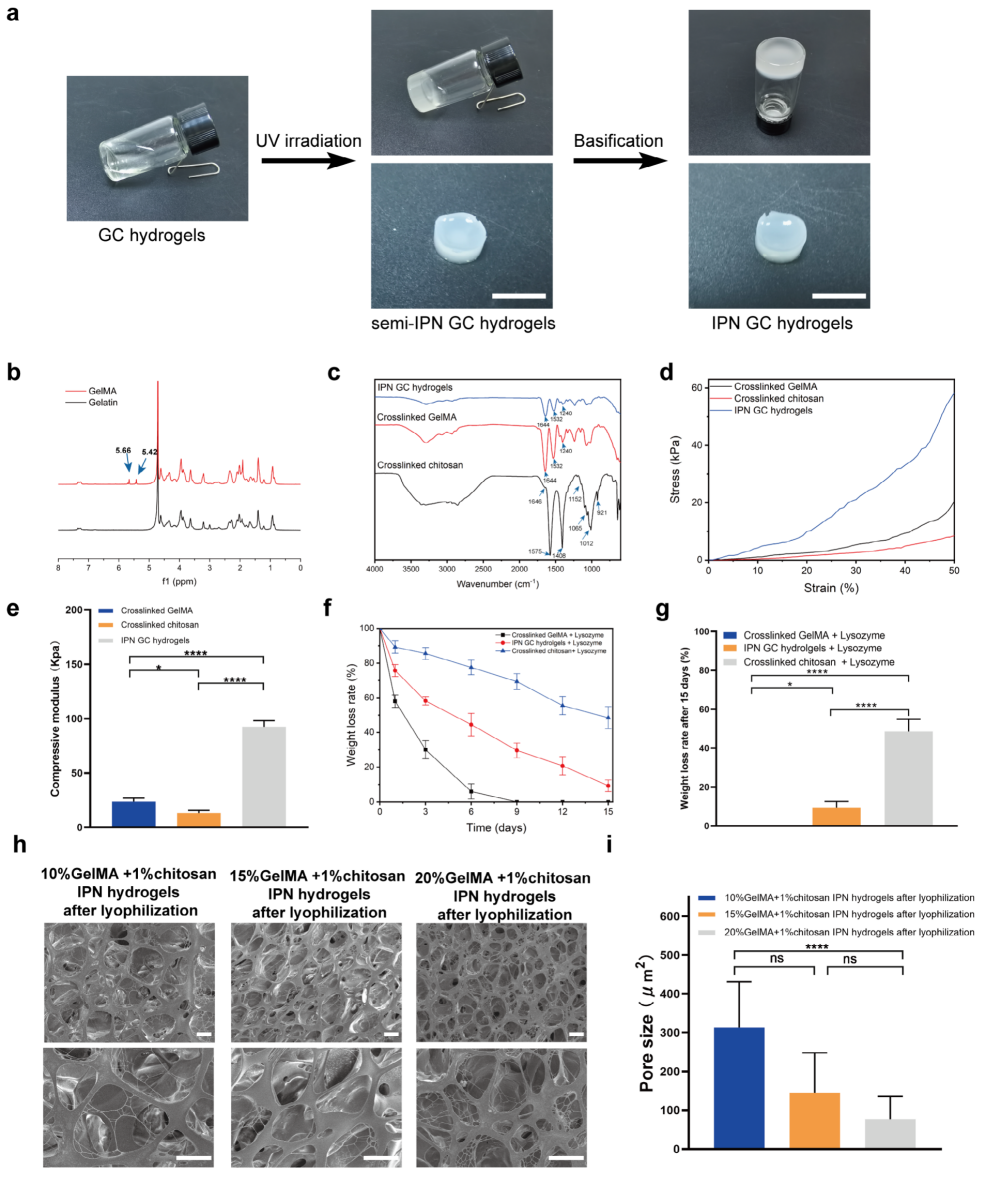
Preparation and characterization of hydrogels. (a) Preparation process of IPN GC hydrogels. Scale bar = 10 mm. (b) ^1^ H NMR spectra of GelMA and gelatin. (c) FT-IR spectrum of crosslinked GelMA, crosslinked chitosan, and IPN GC hydrogels. (d) Compressive stress-strain curves for different hydrogels. (e) Statistical analysis of the compression modulus. (f) Trend in weight loss rate for different hydrogels. (g) Statistical analysis of weight loss rate after 15 d in (f) for different hydrogels. (h) Morphology and surface of different IPN GC hydrogels under SEM at various magnifications after freeze-drying. Scale bar: 10 μm. (i) Statistical analysis of the surface pore size of 10%, 15%, and 20% GelMA + 1% chitosan IPN hydrogels after freeze-drying. ****p < 0.0001; ns, p > 0.05; statistical significance was analyzed using unpaired independent-sample t-test

Initially, GelMA and chitosan were selected as microcarrier biomaterials. According to the properties of these compounds, a photo-crosslinking and basification approach was applied to prepare IPN GC hydrogels (Fig. 2a). UV irradiation covalently crosslinked the GelMA network and chitosan was added as a pH-dependent cationic polymer. The basification process decreased the electrostatic repulsion of protonated NH_3_^+^ and promoted extensive hydrophobic interactions and hydrogen bonding between the chitosan molecules, leading to IPN formation [37]. The IPN structure comprises an entangled and interpenetrated chitosan network and covalently crosslinked GelMA network.

To determine whether gelatin was successfully converted to GelMA, proton nuclear resonance (^1^ H NMR) spectroscopy was employed (Fig. 2b). We observed new signals at 5.42 and 5.66 ppm, corresponding to the two protons of the methacrylate double bond, thus confirming integration of methacrylate into gelatin molecules. Quantitative analysis further indicated that the degree of GelMA methacrylation was 81.3%. In addition, Fourier transform infrared (FT-IR) data revealed that the crosslinked chitosan exhibited adsorption bands typical of the chitosan saccharide structure, including bands at 3300–3500 cm^-1^ corresponding to the partially overlapped amine and hydroxyl stretching vibrations, 1575 cm^-1^ corresponding to amide and amine bending vibrations, 1408 cm^-1^ corresponding to O-H bending vibrations, 1646 cm^-1^ corresponding to amide C = O stretching vibrations of amide I, 921 cm^− 1^ and 1152 cm^− 1^ corresponding to saccharide units of chitosan stretching, as well as at 1012 and 1065 cm^− 1^ resulting from C-O and C-N stretching, respectively [38]. Crosslinked GelMA was characterized by a carbonyl peak at 1644 cm^− 1^ (amide I, caused by C-N stretching vibrations), amino absorption band at 1532 cm^− 1^ (amide II, caused by CH2 wagging vibrations), and a peak at 1240 cm^− 1^ (amide III, caused by amide N-H in-plane bending vibrations) [39]. The FTIR spectra of IPN GC hydrogels were similar to those of crosslinked GelMA, likely due to a high proportion of GelMA in the IPN GC hydrogels [27] (Fig. 2c).

The results of the compression test showed that crosslinked chitosan is fragile with a compressive modulus of only 13.2 ± 2.4 kPa (Fig. 2d), whereas that of the crosslinked GelMA hydrogel is 23.7 ± 3.2 kPa, and the IPN GC hydrogel is 92.4 ± 5.7 kPa. Hence, the IPN structure enhanced the mechanical properties (p < 0.05, Fig. 2e).

After 15 days, the residual weights of crosslinked GelMA, IPN GC hydrogels, and crosslinked chitosan with lysozyme were recorded at 0 ± 0%, 9.3 ± 3.3%, and 48.5 ± 6.3%, respectively (Fig. 2f and g). Hence, the degradation of crosslinked GelMA occurs rapidly; however, the chitosan component, as well as the strengthened networks in the IPN GC hydrogels, effectively reduced this effect.

Further, to observe the surface structure micromorphology of freeze-dried IPN GC hydrogels, scanning electron microscopy (SEM) was performed. Results revealed a nanofiber network structure and a high porosity degree in the surface of 10%, 15%, and 20% GelMA + 1% chitosan IPN hydrogels after lyophilization (Fig. 2 h). More specifically, pore size was found to increase with decreasing GelMA concentration. Indeed, the pore size of freeze-dried 10% GelMA + 1% chitosan IPN hydrogels was significantly larger than that of the 20% GelMA + 1% chitosan IPN hydrogels (p < 0.0001, Fig. 2i). Moreover, as described in previous studies, cell attachment to the hydrogel was dependent on the chemical composition, surface topography, and degree of porosity [12, 15]. Thus, our IPN GC hydrogels prepared with chitosan and GelMA are rich in Arg-Gly-Asp (RGD), contain a nanofiber network comprising a rearrangement and entanglement of chitosan and GelMA chains, as well as adjustable pore size, and are, therefore, capable of improving cell recognition and adhesion [40, 41].

### Design of the T-junction microfluidic chip and scalable production of diameter-controllable IGMs

**Fig. 3:**
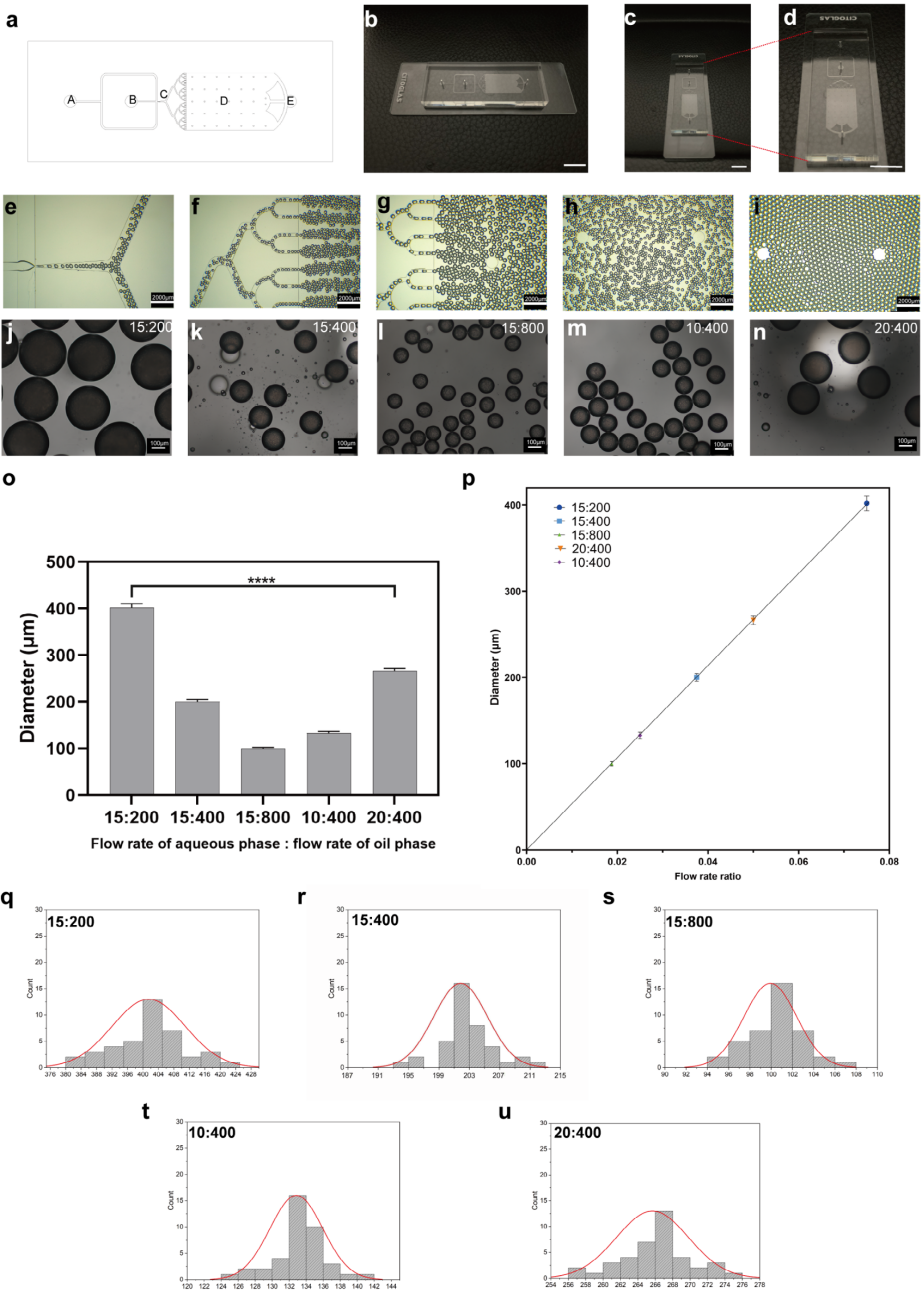
Design of T-junction microfluidic chip and scalable production of diameter-controllable IGMs. (a) Computer-aided design (CAD) blueprint of the T-junction microfluidic chip. (b–d) Photographs of the T-junction microfluidic chip from the side (b), end under low power (c), and end under high power (d). (e–i) Process of droplet formation and flow into the UV irradiation area. (j–n) IGMs with different diameters fabricated by varying the oil and aqueous flow rates. (o) Statistical diagram of IGMs with different diameters resulting from varying the oil and aqueous phase flow rates. (p) Linear relationship between IGM diameters and the flow rate ratio. (q–u) Distribution graphs of microcarrier size adjusted by different oil and aqueous flow rates

Droplet microfluidics is an advanced technology capable of processing and manipulating exceptionally small liquid volumes (10^− 9^ to 10^− 8^ L) in microchannels (tens to hundreds of microns) [42]. Owing to its mass production capacity, as well as its ability to tightly regulate the size, structure, and function of test materials, droplet microfluidics has unique advantages in the preparation of microcarriers with potential biomedical applications [43]. The generation of droplets via microfluidic techniques results from the counterbalance of viscous stress and interfacial tension when two immiscible fluids contact each other in a microfluidic channel [44]. The droplets are then crosslinked to form stable MCs. Herein, we designed and prepared a T-junction microfluidic chip containing two inlets (Fig. 3a-A, a-B), an emulsification compartment (Fig. 3a-C), an UV irradiation area (Fig. 3a-D), and one outlet (Fig. 3a-E). The microfluidic chip was attached to a glass slide (Fig. 3b–d). To prepare the IGMs, GC hydrogels were set as the aqueous (water) phase flowing into inlet b and the oil phase flowing into inlet a; the specific steps to fabricate the IGMs have been described previously [45]. Figure 3e–i shows the process of droplet formation and flow into the UV irradiation area. Moreover, we adjusted the flow rate ratio of the oil and aqueous phases to obtain microcarriers with distinct diameters (Fig. 3j–n). When the oil phase flow rate was fixed at 400 µL/min and the aqueous phase flow rate was varied to 10, 15, and 20 µL/min, the IGM diameters increased from 132.65 ± 2.81 to 201.38 ± 4.45 and 265.67 ± 4.15 μm, respectively (Fig. 3o). Alternatively, when the oil phase flow rate varied at 200, 400, and 800 µL/min, while the aqueous phase flow rate remained constant (15 µL/min), the IGM diameters decreased from 401.48 ± 9.43 to 201.38 ± 4.45 and 99.93 ± 2.47 μm, respectively. Finally, we conducted linear regression analysis based on the flow rate ratio and IGM diameters and observed a linear relationship (Fig. 3p). Hence, the flow rate ratio can be adjusted to accurately control IGM diameter. The size distribution graphs of microcarriers with different flow rate ratios are shown in Fig. 3q–u. The morphology and dispersion of IGMs with five distinct diameters illustrated that the emulsification process was complete, as they exhibited uniform round morphology. Moreover, based on the observed spreading area of the cells, the ~ 200 μm IGMs, which had sufficient space to facilitate the survival and growth of a certain number of DPCs, were used for subsequent experiments [23].

### Compression and swelling properties of IGMs

**Fig. 4:**
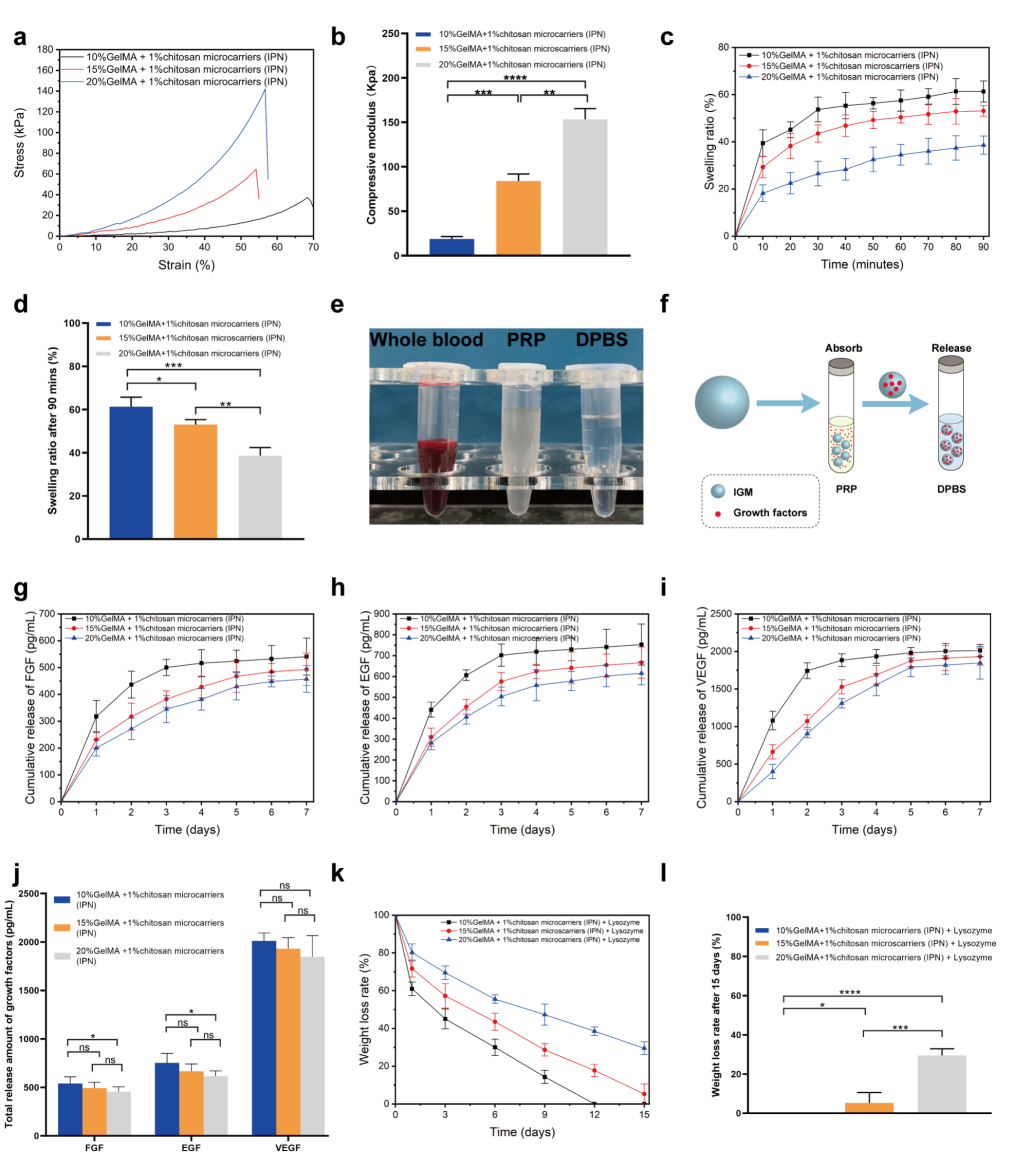
Compression, swelling, growth factor release, and degradation testing of IGMs. (a) Compressive stress-strain curves for different IGMs. (b) Statistical analysis of the compression modulus. (c) Trend in swelling ratio for different IGMs. (d) Statistical analysis of swelling ratio after 90 min in (c). (e) Whole blood, PRP, and DPBS. (f) Schematic of the growth factor release test. Cumulative release of FGF (g), EGF (h), and VEGF (i) for different IGMs. (j) Statistical analysis of total growth factor release amount after 7 d in (g, h, and i). (k) Trend in weight loss rate for different IGMs. (j) Statistical analysis of weight loss rate after 15 d in (k) for different IGMs.

First, we conducted a compressive stress-strain test. The resulting curves showed that 10%, 15%, and 20% GelMA + 1% chitosan microcarriers collapsed at 37.4 ± 3.9, 64.6 ± 5.2, and 142.0 ± 9.8 kPa, respectively (Fig. 4a). Thus, the IGMs were determined to have relatively high mechanical strength, a property that was significantly influenced by GelMA. Additionally, the compression modulus in 10%, 15%, and 20% GelMA + 1% chitosan microcarriers were 18.7 ± 2.8, 84.1 ± 7.9, and 153.2 ± 12.2 kPa, respectively (Fig. 4b). One potential cause of the enhanced stiffness is the substantially reduced space available for the photo-crosslinked GelMA network in the IGM, thus, limiting movement following the embedding of chitosan chains. Moreover, hydrophobic interactions and hydrogen bonding play a pivotal role in enhancing the mechanical strength.

We then investigated the swelling properties of IGMs. First, we immersed the IGMs in Dulbecco’s phosphate buffered saline (DPBS) for 90 min at 37℃, during which the DPBS was gradually encapsulated within the IGMs, as evidenced by their swelling ratios (61.3 ± 4.5%, 53.1 ± 2.2%, and 38.6 ± 3.8%) after 90 min in 10, 15, and 20% GelMA + 1% chitosan microcarriers, respectively (Fig. 4c). After 90 min, the swelling ratio of the IGMs remained relatively unchanged. In addition, statistical analysis reveals that the swelling ratio of IGMs with high GelMA concentration was significantly lower than that of IGMs with a low GelMA concentration (p < 0.05, Fig. 4d). This may be due to the IGMs generated with high concentration GelMA having a denser polymer network and, thus, a smaller molecular gap and reduced swelling ratio. Additionally, based on the results of a previous study, we fixed the chitosan concentration at 1% to ensure that the spreading area of cells was not disturbed [22].

### ***In vitro*** growth factors release from IGMs

Recently, PRP has gained popularity in the treatment of hair loss owing to its ability to boost hair growth and regeneration. We extracted PRP from the whole blood of mice (Fig. 4e) and speculated that IGMs loaded with growth factors could have a relative long actuation duration on hair regeneration. Further, FGF and EGF could improve DPC proliferation and sustain the hair-inductive capacity of DPCs, while VEGF promotes angiogenesis and provide nutrition for novel HFs [27]. We then investigated the release behavior of growth factors in IGMs (Fig. 4f).

To investigate the growth factor release behavior of IGMs, they were placed in DPBS at 37 °C after full absorption. The cumulative release of FGF, EGF, and VEGF from IGMs was evaluated at 1, 2, 3, 4, 5, 6, and 7 d; that of FGF was 540.6 ± 69.0, 493.2 ± 60.5, and 457.1 ± 60.2 pg/mL (Fig. 4 g); that of EGF was 753.2 ± 98.5, 666.2 ± 74.9, and 615.5 ± 55.4 pg/mL (Fig. 4 h), and that of FGF was 2011.8 ± 81.3, 1931.5 ± 114.0, and 1849.0 ± 217.7 pg/mL (Fig. 4i) after 7 d in the 10, 15, and 20% GelMA + 1% chitosan microcarriers, respectively. The total release amount of FGF and EGF in 10% GelMA + 1% chitosan microcarriers was significantly higher than that of 20% GelMA + 1% chitosan microcarriers (p < 0.05, Fig. 4j). This may be due to the increased absorptive capacities of IGMs with higher swelling ratios (lower GelMA concentration/mechanical strength), which might lead to increased uptake of liquid containing growth factors. Moreover, IGMs in all groups exhibited a sustained PRP-release capacity, with the rate of release decreasing after 1 d. Additionally, lower GelMA concentrations corresponded to faster release rates. Overall, 10% GelMA + 1% chitosan microcarriers exhibited superior total release amounts and a higher release rate, whereas 20% GelMA + 1% chitosan microcarriers exhibited a better sustained release rate, while the total release amount was lower.

### Degradation properties of IGMs

Understanding the degradation properties of hydrogels is also necessary to determine whether they can be implanted in humans under clinical applications. Given that lysozyme is widely distributed throughout human tissues, we postulated that the addition of lysozyme to the in vitro degradation test could better simulate the degradation process in vivo. Results show that the degradation curve of the 10%, 15% and 20% GelMA + 1% chitosan microcarriers with 1000 U/mL lysozyme gradually reduced with increased incubation time. After 15 days, the residual weights of the three groups with lysozyme were recorded at 0% ± 0%, 5.3% ± 5.3%, and 29.5% ± 3.3%, respectively (Fig. 4k). Statistical analysis revealed that the degradation time of microcarriers with high GelMA concentration was significantly prolonged compared with that of microcarriers with low GelMA concentration (p < 0.05, Fig. 4 L). However, the degradation properties of 15% GelMA + 1% chitosan microcarriers could be more suitable for HF tissue engineering as the degradation time in this group was similar to that reported for HF formation (2–3 w) [46]. Hence, these IGMs could provide a sufficient period to load DPCs, thus optimizing their induction function. Moreover, these IGMs are completely degraded once the HFs are regenerated.

We then assessed the degradation of 15% GelMA + 1% chitosan microcarriers in vivo and observed negligible amounts of residual IGMs on the 15th day, suggesting their degradation in vivo (Fig. S1). Accordingly, we selected 15% GelMA + 1% chitosan microcarriers for further analysis.

### PRP-loaded IGMs promote DPC proliferation and migration

DPCs play a key role in HF tissue engineering; however, variations in the DPC phenotype hinder their application in clinical practice. Previous studies have reported that growth factors can promote DPC proliferation while retaining their DP signature [6]. We sought to determine a suitable PRP concentration that includes sufficient levels of growth factors to promote the physiological process of DPCs. Therefore, we diluted PRP to 10%, 20%, and 30% and loaded them into IGMs (IGMs + 10% PRP, IGMs + 20% PRP, and IGMs + 30% PRP respectively). These IGMs, as well as the control (IGMs + 0% PRP) were added to DPC cultures to observe and compare cell proliferation, migration, and DP signatures. DPCs treated with IGMs + 20% PRP had the highest percentage of Ki67^+^ cells—a biomarker of proliferation—(Fig. S2a and b). Moreover, flow cytometric analysis revealed that the highest proportion of DPCs treated with IGMs + 20% PRP entered S phase of the cell cycle (p < 0.05) (Fig. S2c and d). The S phase (i.e., DNA synthesis stage), refers to the period from the beginning to the end of DNA synthesis. During this period, DNA replication and transcription, as well as protein synthesis, are completed. Hence, more cells in S phase indicates active cell division [47]. In addition, CCK-8 was performed to assess DPC proliferation revealing that the number of DPCs increased with increasing culture time and was significantly larger in IGMs + 20% PRP compared with the other groups after 36 h (p < 0.05, Fig. S2e). In vitro migration assays revealed that the cells in the IGMs + 20% PRP group exhibited the highest migration rate (~ 80% after 36 h) while those in the other groups did not exceed 72% (Fig. S2f and g). Furthermore, we explored the expression of DP signatures (verscian, NCAM, β-catenin, and ALP) associated with HF inductivity in the four groups. Western blotting revealed that versican expression in the IGMs + 20% PRP group was significantly higher than in the IGMs + 0% and 30% PRP groups, while no significant differences were observed compared with the IGMs + 10% PRP group. The expression of NCAM, β-catenin, and ALP in the IGMs + 20% PRP group was significantly higher than in the IGMs + 0% and 10% PRP groups, while no significant differences were observed with the IGMs + 30% PRP group (Fig. S2h and i). Q-PCR results confirmed these findings (Fig. S2j, k, l and m). Through comprehensive comparison, the IGMs + 20% PRP group was determined to have the strongest HF inductivity.

Collectively, these results indicate that IGMs + 20% PRP most effectively trigger DPC proliferation, migration, and HF induction ability, while the therapeutic effect of platelets contained in PRP increases with an increase in concentration, within a given range, which is consistent with a previous report [48].

### PRP-loaded IGMs enhance the therapeutic effects of PRP in transition from telogen to anagen

We next sought to evaluate the effect of 20% PRP-loaded IGMs on hair growth and the hair cycle compared with that of 20% PRP direct injection and DPBS (control group) in vivo. To this end, we preformed depilation of dorsal skin to synchronously induce each mouse to initiate the hair cycle from the telogen to anagen phase, as described previously [49]. Mice were subcutaneously injected with 20% PRP-loaded IGMs or 20% PRP and DPBS (Fig. S3a). Subsequently, based on the findings of a previous study [50], we assessed mouse skin color and the relative area of anagen HFs to analyze the level of hair growth. That is, since the HF melanogenic activity correlates with hair growth, murine skin color gradually changes with accumulation of melanin from pink to dusky pink, grayish pink, grayish, grayish black, and finally black. Meanwhile, the color of depilated skin is closer to pink during the telogen phase and black during the anagen phase [37]. Therefore, we created a scoring system, in which each color was assigned a specific score that represented a sub-phase of the hair cycle (Fig. S3b).

The color change of depilated skin in the 20% PRP-loaded IGM group occurred significantly faster than that in the other two groups from day 0 to day 9 (p < 0.05, Fig. S3c and d). Hence, the 20% PRP-loaded IGMs group experienced accelerated transformation from the telogen to anagen phase. Thirteen days following depilation, no difference in skin color scores was observed among the three groups, indicating that they had all entered the anagen phase. However, after entering the anagen phase, the 20% PRP-loaded IGM group exhibited significantly more novel hair growth (p < 0.01) in the depilation area compared to the other groups at day 13. Moreover, the 20% PRP group showed more new HFs than the control group (p < 0.01; Fig. S3c and e).

On days 5, 9, and 13, hematoxylin-eosin (HE)-stained patches of dorsal skin from the three groups were examined (Fig. S3f). On day 5, the HFs demonstrated the partial onset of melanogenesis. Further, DP with a markedly large diameter present at the deeper dermis was observed in the mice from the 20% PRP-loaded IGMs group, compared to those from the mice in the other groups, indicating that they had entered the anagen II–III phase; the mice from the other two groups remained in the telogen phase (DP with a smaller diameter present in the dermis). Therefore, the 20% PRP-loaded IGMs group enhanced the therapeutic effects of PRP in the transition from telogen to anagen. Moreover, on day 9, the HFs in the mice from the 20% PRP-loaded IGMs group were determined to have entered the anagen V–VI phase (narrow DP in the deep subcutis above the panniculus carnosus, and the tip of the hair had entered the hair canal or emerged through the epidermis), while the HFs in the control and 20% PRP direct injection groups had entered the anagen I–II phase (round, compact ball-like DP located in the dermis, and a thickening and prolongation of the keratinocyte strands) and anagen III phase (DP located at the border between the dermis and subcutis and almost all HFs had begun to demonstrate melanogenesis), respectively. Hence, hair was found to first protrude from the skin in the mice of the 20% PRP-loaded IGMs group. Meanwhile, by day 13, the mice from all three groups had entered the anagen V–VI phase, where they would remain for several days and gradually enter the catagen phase [49].

Collectively, these data indicate that 20% PRP can accelerate hair cycle transformation from the telogen to anagen phase. Given that all variables were held constant, save for release speed (actuation duration), between the 20% PRP direct injection and 20% PRP-loaded IGMs groups, we consider that the differences in the observed phenomena were attributed to the sustained (rather than burst) release of PRP in 20% PRP-laden IGMs. Indeed, reduction of the growth factor release rate resulted in a reduced subcutaneous tissue absorption rate and maintenance of effective growth factor concentrations for an extended period of time in vivo, thus prolonging the therapeutic effect.

### Cellular biocompatibility of DPCs co-cultured with IPN GC hydrogels and IGMs

Previous studies have illustrated that DP isolated from mice can induce HF formation when implanted into recipient non-hairy skin, suggesting that DP can reprogram non-hairy epidermis to a follicular fate [51]. Thus, an HF tissue engineering approach will require extensive inductive DPCs. Moreover, DPCs as ADCs with no attachment to a substrate cannot readily survive. Our supplemental data confirmed that the viability of DPC aggregates without suitable substrate was < 30% on day 7 in a low adhesion culture plate (see Fig. S4a, b). Similarly, Chen et al. demonstrated that spheroidal ADCs are induced to undergo apoptosis, while stretched cells can survive and proliferate [23]. In addition, hypoxic conditions within the center of aggregates induce cell death [12]. Therefore, we chose to seed DPCs on the surface of IGMs.

**Fig. 5:**
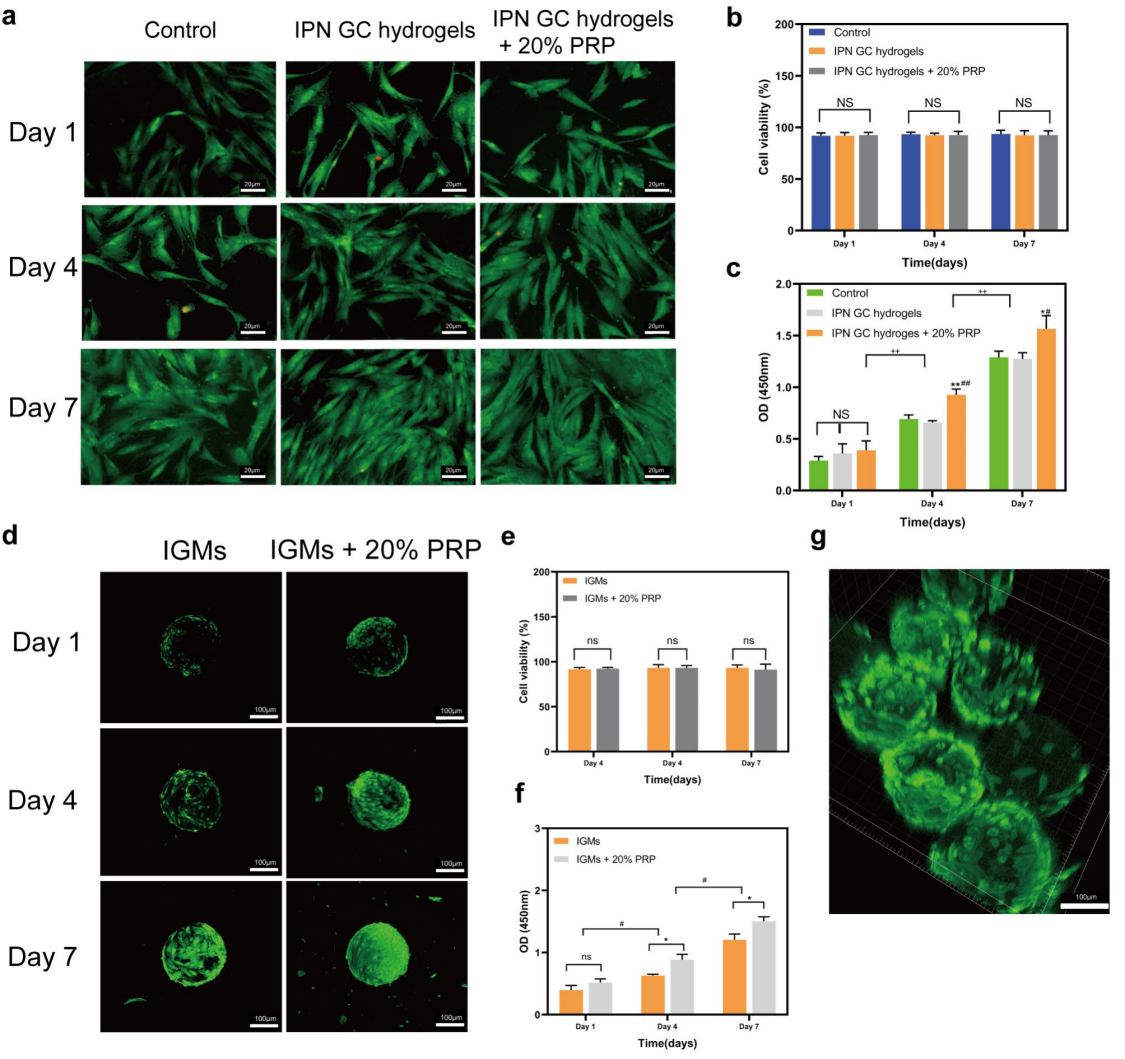
Cellular biocompatibility of DPCs co-cultured with IPN GC hydrogels and IGMs. (a) DPCs for three groups (green represents live cells, and red indicates dead cells). (b) Statistical diagram of cell viability % for DPCs (NS, no significance). (c) Statistical diagram of cell proliferation activity (*p < 0.05; **p < 0.01 IPN GC hydrogels + 20% PRP relative to the control group; #p < 0.05; ##p < 0.01 IPN GC hydrogels + 20% PRP relative to IPN GC hydrogels; ++p < 0.01, day 7 relative to day 1 and day 4). (d) DPCs seeded on IGMs and IGMs + 20% PRP for 1, 4, and 7 days detected by live/dead staining. Statistical diagram of cell viability (e) and cell proliferation activity (f) of DPCs in two groups (*p < 0.05 related to IGM; **p < 0.01 related to IGMs; #p < 0.05, day 7 relative to day 1 and day 4). (g) 3D confocal fluorescence microscopy images showing the mutual adhesion of IGMs (red represents DPCs). Statistical significance was analyzed using a one-way ANOVA for three groups and unpaired independent-samples *t*-test for two groups; n = 3 for each group

First, DPCs were divided into three groups: control (DPCs seeded on the tissue culture polystyrene [TCP] without additives), IPN GC hydrogels (DPCs co-cultured with IPN GC hydrogels), and IPN GC hydrogels + 20% PRP (DPCs co-cultured with IPN GC hydrogels and 20% PRP was added into culture). Live/dead staining was performed to detect cell viability. Most DPCs among the three groups stained green (alive) on days 1, 4, and 7 (all > 90%; Fig. 5a, b), with no significant differences in viability among the three groups. In addition, CCK-8 was performed to assess DPC proliferation. The number of DPCs increased with increasing culture time and was significantly larger in IPN GC hydrogels + 20% PRP compared with the other groups at days 4 and 7 (p < 0.01), while no differences were observed on day 1 (p > 0.05, Fig. 5c).

Furthermore, cell viability and the proliferative activity of DPCs seeded on IGMs and IGMs + 20% PRP (20% PRP-loaded IGMs) was assessed. Confocal images showed that most DPCs in the two groups were alive on days 1, 4, and 7 (both > 90%; Fig. 5d) with no significant differences between the groups (Fig. 5e). Moreover, DPCs on IGMs + 20% PRP exhibited higher proliferative activity than the IGMs group on days 4 and 7 (Fig. 5d, f). Considering that the DPCs in the IGM group showed partial spreading, while those in the IGMs + 20% PRP group exhibited uniform and complete spreading on day 4, we concluded that PRP can promote DPC spreading. Additionally, a portion of the IGMs became interconnected, thus forming a self-assembled structure due to the mutual attraction and adhesion between cells, providing an appropriate 3D-culture environment for DPCs (Fig. 5 g).

Collectively, these two natural biomaterials were found to provide appropriate microenvironments for DPCs, including an ECM, which is the basis for cell bioactivity [22]. Additionally, these results indicate that the IPN network of GC hydrogels could increase the stiffness of IGMs to provide mechanical support for DPCs and enhance cell adhesion, spreading, and related behaviors [23]. Finally, the ability of IGMs to sustain the release of PRP also plays a critical role in promoting DPC proliferation.

### Morphological analysis and DP signature expression of DPCs on IGMs

**Fig. 6:**
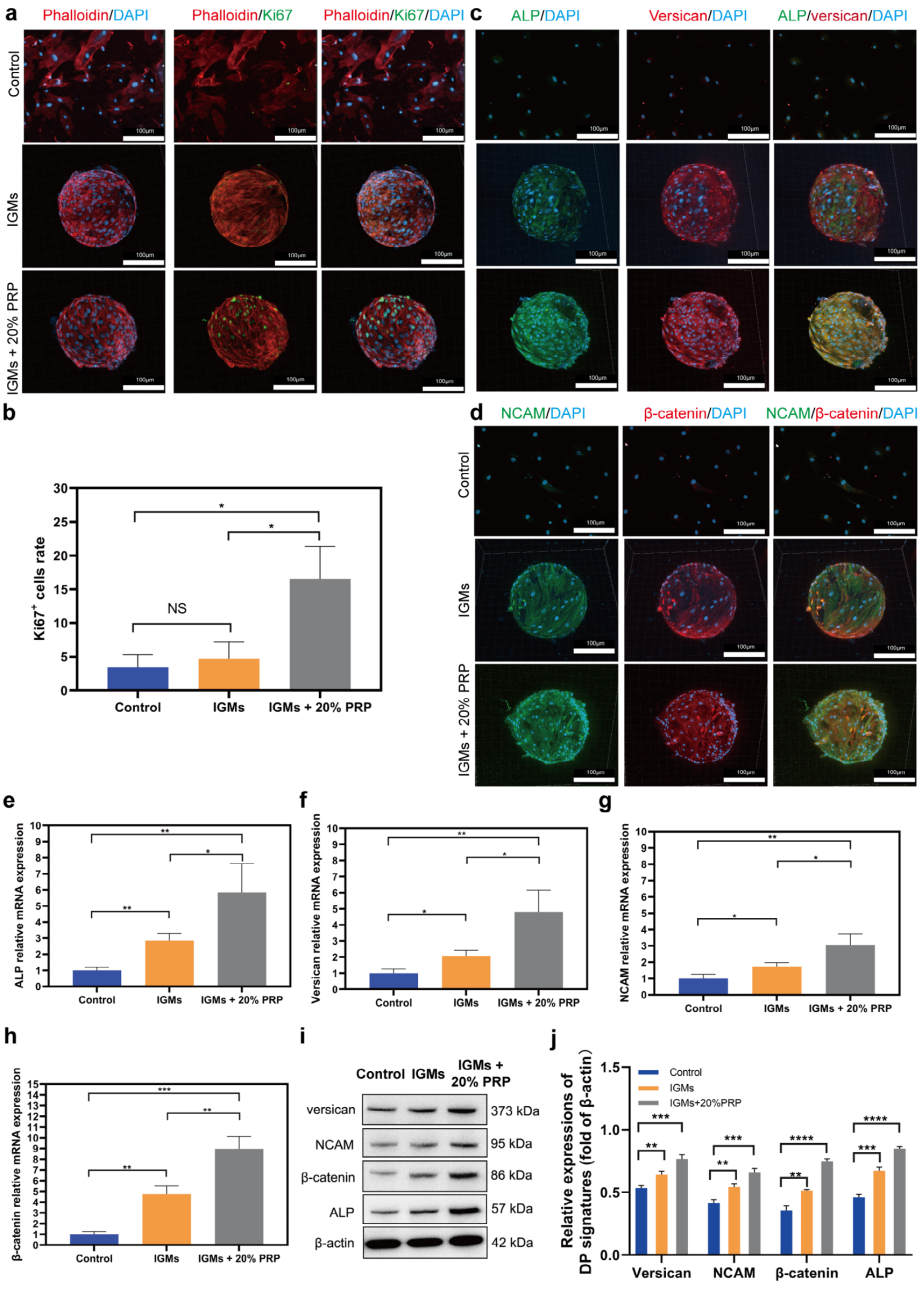
Morphological analysis and expression of DP signatures of DPCs on IGMs: (a) 2D and 3D confocal images of DPCs stained by Phalloidin and Ki67 for three groups. (b) Statistical analysis of the number of Ki67^+^ DPCs in three groups. Immunofluorescence staining images (c, d), Q-PCR (e, f, g, h) and western blotting assays (i, j) showing the expression of ALP, versican, NCAM, and β-catenin among the three groups; *p < 0.05; **p < 0.01; statistical significance was analyzed using unpaired independent-samples t-test

To further investigate DPC morphology and intuitively show DPC proliferation among the groups of control (DPCs seeded on the TCP), IGMs, and IGMs + 20% PRP, DPCs were stained with phalloidin-TRITC, Ki67, and DAPI to visualize F-actins, proliferative cells, and nuclei after a 7-day culture (Fig. 6a). The morphology of all three groups exhibited good cell adhesion and spreading. Moreover, 3D images showed that the distribution of DPCs on IGMs and IGMs + 20% PRP was relatively uniform and that DPCs adhered to the surfaces of IGMs. Importantly, the focal adhesion of DPCs on IGMs was successfully illustrated. For DPCs as a type of ADC, a spread morphology associated with focal adhesion is directly conducive to maintaining their original phenotype and functionality. Otherwise, the trans-differentiation and apoptosis pathways could be easily activated [52]. Indeed, F-actin, which enables and accelerates the orientation of the cytoskeleton and ultimately spreads throughout the cell body, is a crucial marker to confirm that IGMs are conducive to promoting focal adhesion [53].

Ki67 staining revealed that the IGMs + 20% PRP group contained the most Ki67^+^ cells, while the control group had the fewest. Statistical analysis was consistent with immunofluorescence staining images (Fig. 6b), indicating that PRP loaded in IGMs exhibited the ability to promote DPCs to enter the active phase of the cell cycle.

Furthermore, we explored the expression of DP signatures (ALP, verscian, NCAM, and β-catenin) associated with HF inductivity in the three groups. Immunofluorescence staining images revealed that the expression of all four proteins was highest in the IGMs + 20% PRP group. Moreover, their abundance was greater in the IGM group than in the control group (Fig. 6c and d). Subsequent q-PCR (Fig. 6e–h). and western blot results confirmed these findings (Fig. 6i and j). Supplemental data confirmed that DPCs within aggregates exhibited reduced DP marker expression from day 1 to 7 (see Fig. S5). This may have been caused by apoptosis and the absence of focal adhesion. Taken together, these data indicate that IGMs + 20% PRP have the ability to maintain or promote the expression profile of DP signatures to enhance hair inductive properties of DPCs in vitro.

Further, IGM diameter might be related to hair inductive properties or the efficiency of hair regeneration. On the one hand, in a unit volume, the smaller the IGM diameter, the larger the available specific surface area to carry DPCs. Indeed, the number of DPCs were positively correlated with hair induction intensity. On the other hand, excessively large IGMs are not conducive to injection as they may cause blockage or increased mechanical shear stress during injection, potentially leading to cell membrane damage [54]. However, given that cell shape reportedly governs whether cells grow or die [23], ADC adhesion and spreading is hampered by relatively small substrate areas, leading to reduced viability and proliferation. Hence, while it is necessary to retain a small IGM diameter, it is also vital that DPCs be able to adhere and stretch normally. Our study found that ~ 200 μm IGMs are suitable for HF tissue engineering; however, we did not assess the impact of IGMs with smaller diameters on HF induction.

Given that DPC size is fixed, the diameter of IGMs impacts the curvature of the cell substrate. Importantly, convex surfaces significantly impact the cell cycle and cytoskeleton mediated via the BAR (Bin/amphiphysin/Rvs) domain proteins [55]. In addition, on convex surfaces, cytoskeletal forces lead to substantial nuclear deformation, increased lamin-A levels in MSCs, and promotion of osteogenic differentiation [56]. Moreover, RNA-Seq analysis of MSCs on the sinusoidal curvature platform and flat surface identified differentially expressed genes related to differentiation processes, cytoskeleton remodeling, and cell proliferation [57]. However, to date, the effect of cell substrate curvature on DPC behavior and hair regeneration efficiency remains unclear and warrants further investigation.

### PRP-loaded DECHS improves HF inductive ability

To date, several hair regeneration protocols (models) have been established and are represented by the ‘‘patch assay”, in which epithelial and dermal cell mixtures are injected into subcutaneous space of immunodeficient mice, [58] and the ‘‘chamber assay” comprising epithelial and dermal cell mixtures transplanted into a silicone chamber grafted on the back of immunodeficient mice [59]. Additionally, the “Flap assay” and “sandwich model” can be used to evaluate the hair inductive ability of DPCs [58, 60]. Owing to its simple operation, fewer complications, and biomimetic environment, the “patch assay” is more commonly used in hair regeneration models compared to the other protocols and was, therefore, adapted in the current study.

**Fig. 7:**
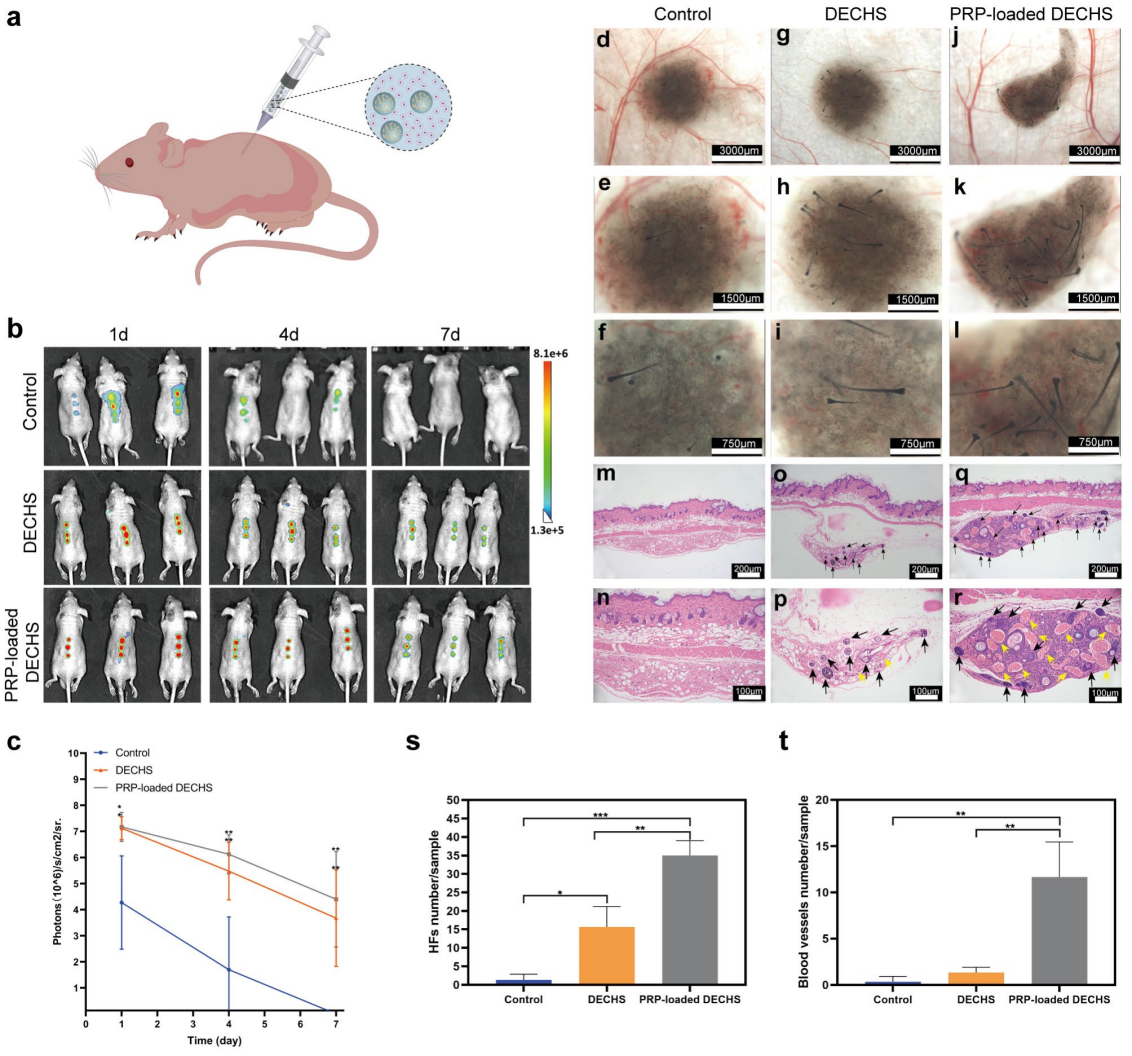
PRP-loaded DECHS improves the HF inductive ability. (a) Schematic diagram of injecting PRP-loaded DECHS into a nude mouse; (b) in vivo fluorescence imaging following subcutaneous injection of DIO-labeled cells in the control group, DECHS group, and PRP-loaded DECHS group. (c) Quantitative diagram of the DIO signal intensity expressed as photons/s/cm^2^/sr. Stereomicroscopy images of injection sites in the control group (d–f), DECHS group (g–i), and PRP-loaded DECHS group (j–l). HE-stained images of the injection site in the control group (m, n), DECHS group (o, p), and PRP-loaded DECHS group (q, r). Black arrows: regenerative HFs, yellow: regenerative blood vessels. Statistical diagrams of HF numbers/sample (s); and blood vessel numbers/sample (t) among the three groups. *p < 0.05; **p < 0.01; ***p < 0.001; statistical significance was analyzed using unpaired independent-samples t-test

To observe the DPCs in subcutaneous tissue in real time, DPCs were tagged with 3,3′-dioctadecyloxacarbocyanine perchlorate (DIO) before injection. DPC-laden IGMs with or without 20% PRP were mixed into EPC-laden GelMA to establish a DECHS and PRP-loaded DECHS. Subsequently, DECHS and PRP-loaded DECHS groups were transplanted subcutaneously into nude mice (Fig. 7a) and compared with the outcome of a conventional approach (control), described in a previous study [4]. In vivo fluorescence imaging indicated that more DIO-labeled cells were localized at the injection site in the DECHS and PRP-loaded DECHS groups after 7 days compared to the control group, in which DIO-labeled cells were dispersed throughout the subcutaneous tissue 1 day after injection and were undetectable after 7 days (Fig. 7b). Moreover, statistical analysis revealed good retention of living DIO-labeled cells in the DECHS and PRP-loaded DECHS groups, which would support HF regeneration and formation (Fig. 7c). Indeed, 17 days after implantation, stereomicroscopic images showed a small number of novel HFs regenerated in the control group (Fig. 7d–f), moderate amounts of new HFs in the DECHS group (Fig. 7 g–i), and substantial regenerative HFs in the PRP-loaded DECHS group (Fig. 7j–l).

HE-stained images also showed minimal HF regeneration in the control group under low (10×) and high (20×) magnification (Fig. 7 m and n). In contrast, nine novel HFs and two blood vessels were observed in a hematoxylin-eosin (HE) stained tissue section of the DECHS group (Fig. 7o and p), while 15 regenerative HFs and more than ten novel blood vessels were detected in the PRP-loaded DECHS group (Fig. 7q and r). Statistical analysis confirmed the presence of significantly more HFs and blood vessels in the PRP-loaded DECHS group (Fig. 7s and t). Further, Fig. S6a shows that DPC aggregates cultured for 7 days in low adhesion plates and mixed with EPCs did not readily induce HFs due to perpetual loss of DPC activity over time. Fig. S6b also reveals that DPC/PRP-loaded IGMs cannot induce HF regeneration owing to the lack of epithelial-mesenchymal interactions [61].

In summary, these data indicate that PRP-loaded DECHS not only provides a superior environment for retaining live DPCs, but also for preventing their dispersion to surrounding tissues. Regarding HF regeneration, the IGMs in DECHs provide cell–material interactions, which determine survival/apoptosis, adhesion/migration, proliferation/differentiation, as well as numerous other DPC functions [62]. The efficiency of HF regeneration depends, to a large extent, on the DPC phenotype. Successful settlement of DPCs begins with the binding of their integrin receptors to specific motifs in the ECM. Integrins as transmembrane proteins can recognize and bind Arg-Gly-Asp (RGD), which is abundant in GelMA hydrogel, and subsequently initiate a series of intracellular events, including focal adhesion. Focal adhesion has a considerable impact on transferring survival signals from the ECM via the PI3K-Akt pathway, and impeding the activation of apoptotic proteins (Caspase-9 and p53) [63], while also regulating the dynamic process of cell migration. Moreover, focal adhesion kinase plays a key role in transferring cell proliferation signals to the nucleus [64]. Cell spreading and shape can also be regulated through integrins and focal adhesion activating Rho-family proteins [64, 65]. Hence, IGMs in DECHS ensure a myriad of important DPC behaviors.

PRP-loaded DECHS appeared to reconstruct the signal interactions between DPCs and EPCs to facilitate sustained HF inductive capacity. The signals or pathways connecting the epithelial and mesenchymal components primarily comprise Wnt molecular (*Lef1, Wnt10b* and *Wnt5a*), sonic hedgehog, and Edar signaling genes, as well as BMP signaling inhibitors, such as noggin, which have pivotal roles in HF induction [66]. Additionally, the presence of a considerable amount of PRP growth factors, such as FGF-7/10, TGF-β2, EGF and VEGF, may be crucial to HF tissue engineering. FGF-7/10 and TGF-β2 impact dermis components to enhance HF maturation, while TGF-β2 and EGF can act on hair matrix cells to boost their proliferation [66]. Meanwhile, VEGF may promote the construction of novel vessels that can connect to host vessels in subcutaneous tissue. Generation of new blood perfusion provides nutrition and oxygen to the novel HF tissues to increase hair induction efficiency. Thus, PRP-loaded DECHS offers certain advantages to HF regeneration.

In addition, it is more difficult for human adult DPCs to induce HF regeneration than mouse DPCs, though many common key genes have been identified (e.g. FGF-7, IGF-1, and Wnt-5a), and a similar fundamental molecular mechanism has been reported in DPCs derived from humans and mice to induce novel HFs [67]. Specifically, significantly fewer HFs were induced by human adult-derived DPCs than by mouse DPCs, owing to species-specific differences [68]. Therefore, another limitation of the current study is that human cells and human-derived PRP were not included, which might warrant further investigation to achieve clinical translation. In addition, considering that the low induction ability of human DPCs may not be conducive to HF regeneration, dermal sheath cells might represent an alternate cell type to verify the clinical applicability of our strategy, as the dermal sheath is not only structurally continuous and functionally similar to DP, but can also upregulate trichogenic gene expression [5]. Alternatively, skin-derived precursor cells—another population of HF-related multipotent dermal cells—and umbilical cord-derived MSCs, which have been confirmed to show trichogenic activities when co-cultured with DPCs, may also represent potential cell sources [69]. Moreover, it is necessary to conduct co-culture and differentiation induction studies, to optimize the protocol required to transform multipotent cells into those with DP function.

## Conclusion

In our study, we utilized IPN GC hydrogels to prepare large-scale monodispersed, and diameter-controlled IGMs using droplet microfluidic technology. Additionally, we extracted PRP containing abundant growth factors from mice blood. Based on excellent swelling, sustained release, and degradability properties, IGMs were used to load and sustain the release of PRP to effectively promote the in vivo transition from the telogen to anagen phase of the hair cycle. Moreover, the suitable mechanical properties and biocompatibility of 20% PRP-loaded IGMs improved cell adhesion, spreading behavior, proliferation, and maintenance of the DP signature expression. Importantly, 20% PRP-loaded IGMs were mixed with EPC-laden GelMA to form a PRP-loaded DECHS, which was injected into mice to achieve successful HF reconstruction. Compared to that with the conventional approach, the number of in vivo regenerative HFs and blood vessels increased substantially in the 20% PRP-loaded DECHS group, revealing that the PRP-loaded DECHS promotes hair regeneration efficiency. The 20% PRP-loaded IGMs provide a suitable microenvironment rich in ECM and growth factors to promote DPC proliferation, while retaining the DPC HF inducing ability. The EPC-laden GelMA hydrogel shell is similar to the in vivo epidermal sheath (outer root sheath and inner root sheath) containing EPCs and EPC-related signals. Therefore, the PRP-loaded DECHS also simulates an HF macroenvironment to reconstruct the epithelial-mesenchymal interactions. Hence, our novel strategy could have important applications in HF tissue engineering.

## Methods

### Animals

Male adult athymic nude mice (age: 3–5 weeks), C57BL/6J newborn mice, and 7-week-old C57BL/6J male mice were obtained from the Experimental Animal Center of Southern Medical University.

### Preparation of GelMA hydrogel

GelMA polymer was synthesized according to the procedure reported by Yue et al. [70]. In brief, 10% w/v GelMA was prepared with 5 g of porcine skin-derived powdered gelatin (porcine skin source, analytical grade, 99% pure, Sigma-Aldrich, Saint Louis, USA) dissolved in 50 mL of Dulbecco’s phosphate buffered saline (DPBS, Solarbio, Beijing, China) for 10 min with continuous stirring at 60 °C. Next, methacrylic anhydride (MA, 94%, Sigma-Aldrich) solution was slowly injected dropwise at a flow rate of 0.5 mL/min while on a magnetic stirrer at 50 °C; the volume of the injected MA solution was 10 mL. After the mixed solution was allowed to fully react for 3 h, 250 mL of DPBS was added to the solution to stop the reaction by diluting the mixed solution. Subsequently, the diluted solution was transferred to a dialysis tubing (DPBS, Solarbio) with a molecular weight cut-off of 5.0 kDa and dialyzed in deionized water for one week to completely remove the unreacted reagents. After 7 days, the dialyzed solution (ThermoFisher, Lenexa, Kansas, USA) was centrifuged at 200 × *g* for 6 min to remove impurities until the solution was in a clear liquid state. The supernatant was collected and placed in a freezer (ThermoFisher) at **− 8**0 °C to induce rapid freezing into a solid state. The water in the mixed solution was removed using a freeze dryer (Martin Christ company, Osterode, Germany) for 3 days, and the prepared GelMA polymer was collected as a white polycellular foam stored in a vacuum drier (Yiheng Instrument Co., Ltd, Shanghai, China) until further use.

### IPN GelMA/chitosan (GC) hydrogel preparation

DPBS (6 mL) was added to 900 mg of GelMA. The GelMA polymer solution was stirred for 1 h until it became clear at a final concentration of 150 mg/mL. Next, acetic acid (anhydrous, 99.9%, Aladdin Biochemical Materials Technology Co., Ltd, Shanghai, China) was added to the GelMA solution and evenly mixed to a concentration of 2% v/v. Next, chitosan powder (200–400 mPa s, analytical grade, 99% pure, Aladdin Biochemical Materials Technology Co., Ltd) was added to the mixed solution and placed on a constant temperature magnetic stirrer (Yuhua Instrument Equipment Co., Ltd, Shanghai, China) set at 40 °C for 12 h to dissolve the chitosan to a concentration of 10 mg/mL. Subsequently, 0.5% w/v 2-hydroxy-4-(2-hydroxyethoxy)-2-methylpropiophenone (Irgacure 2959, Sigma-Aldrich) was added to the solution. Finally, we used 365 nm UV light (Zigu Lighting Appliance Factory, Zhongshan, China) to photo crosslink the solution for 30 s before adding it to a 0.5 M sodium hydroxide solution (NaOH, analytical grade, 99% pure, Aladdin Biochemical Materials Technology Co., Ltd) for 20 s to form an IPN GC hydrogel.

### Characterization of IPN GC hydrogels by ^1^ H NMR, FT-IR, and scanning electron microscopy (SEM)

An ^1^ H NMR system (Bruker, Berlin, Germany) was used to characterize the chemical structures of gelatin and GelMA to determine the degree of methacrylation of the free amine group in GelMA. We dissolved 5 mg of gelatin and GelMA in D_2_O (Sigma-Aldrich) and then performed NMR spectroscopy at 50 °C and 600 MHz. The degree of methacrylation was analyzed using Eq. 1:

degree of methacrylation (DM) = I5.27/(I5.27 + I2.84) × 100% (1).

Fourier transform infrared spectroscopy (FTIR) spectroscopy (ThermoFisher) was performed for crosslinked GelMA, crosslinked chitosan, and IPN GC hydrogels to identify their chemical structures. For each spectrum, 64 scans were collected with a resolution of 4 cm^− 1^.

The microscopic morphology and surface of freeze-dried IPN GC hydrogels were observed with a scanning electron microscope (SEM, Hitachi, Ibaraki ken, Japan). In addition, statistical analysis of the freeze-dried IPN GC hydrogel pore size was performed. In brief, 20 pores/per group were randomly selected, the area of each pore was measured using image Pro-Plus 6.0 software.

### Design of the T-junction microfluidic chip

The structure of the microfluidic chip included two inlets (one for the water phase and one for the oil phase), an emulsification compartment, UV irradiation area, and an outlet. The T-junction microfluidic chip was prepared in PDMS according to the soft lithography method [71], by Wuhan Mesoscopic Biotechnology Co., Ltd. The process of droplet production is presented in Addition file 2 and 3 in the microfluidic chip.

### Fabrication of IGMs

Fabrication of IGMs was based on the microfluidic chip technology. The aqueous phase comprised a solution containing GelMA (100, 150, or 200 mg/mL), chitosan (10 mg/mL), 0.5% w/v Irgacure 2959, and 2% v/v acetic acid. The oil phase was composed of mineral oil (anhydrous, ≥ 99.9%, Aladdin Biochemical Materials Technology Co., Ltd) and 5% v/v Span 80 (Aladdin Biochemical Materials Technology Co., Ltd). Two syringes, one containing the aqueous phase solution and the other, the oil phase solution, were placed horizontally on the injection pump (Xunfei Scientific Instruments Co., Ltd, Suzhou, China). The aqueous phase and oil phase solutions were pushed into two separate inlets at distinct flow rates so that water-in-oil droplets were formed and photo crosslinked in a UV irradiation area using a 365 nm handheld UV lamp (Zigu Lighting Appliance Factory) for 20 s before being allowed to flow into a 15-mL centrifuge tube (Corning, New York, USA) containing 1 mL of 0.5 M NaOH, to solidify the chitosan. The obtained IGMs were triple-washed with deionized water and centrifuged at 200 × *g* for 6 min to remove the excess solution as well as the mineral oil and Span 80. Ethanol (Solarbio, Beijing, China) was used to gradually dehydrate the IGMs. Finally, the sample was dried with a scC02 desiccator (Leica, Wetzlar, Germany) for 2 h and stored in a dryer.

### Preparation of activated PRP

PRP was prepared from the blood of C57BL/6 mice using the double-spin method as described by Osada et al. [72]. In brief, the abdomen and peritoneum of the anaesthetized mice were opened with lateral incisions, and the inferior vena cava was exposed after moving the intestines to the other side. Subsequently, a 1-mL syringe (Yibaishun Technology Co., Ltd., Shenzhen, China) containing 100 µL acid-citrate-dextrose (ACD, Sigma-Aldrich) was used to insert the needle into the vena cava and withdraw blood, which was decanted into a 1.5-mL collection tube (see Fig. S7a). Next, 200 µL of the prepared modified Tyrode’s buffer (Kulaibo Technology Co., Ltd., Beijing, China) was mixed with the blood, and the solution was centrifuged at 300 × *g* for 4 min (see Fig. S7b). The upper PRP layer and top third of the lower red blood cell layer were transferred into another 1.5 mL collection tube (the right tube) as shown in Fig. S7c. The collected blood was again centrifuged at 200 × *g* for 6 min (see Fig. S7d). The upper layer (PRP) and the top of the lower red blood cell layer in the middle tube were collected into a new tube (see Fig. S7e, the right tube). Then, an additional 200 µL of the prepared modified Tyrode’s buffer was added to the remaining lower red blood cell fraction of the middle tube and centrifuged at 200 × *g* for 6 min. The same layer from the middle tube was drawn and again added to the tube shown on the right. In addition, the collected blood (shown in the tube on the right in the figure) was centrifuged at 1,000 × g for 6 min (see Fig. S7f). The plasma was aspirated from the pelleted platelets, and the pellet was resuspended in 200 µL of modified Tyrode’s buffer (see Fig. S7g, the middle tube). The platelets were counted using an automatic animal blood cell analyzer (Mindray BC-5000vet, Shenzhen, China) and found to be ~ 2.4 × 10^8^/mL per mouse. Moreover, 10% (w/v) calcium chloride (Sigma-Aldrich) and 1,000 U of bovine thrombin (Sigma-Aldrich) were mixed in a 1:1 (v/v) ratio to prepare a solution that acted as an activator; this solution was added to the PRP solution (10:1 (v/v)). Finally, the mixture was incubated at 23 °C for 15 min to activate PRP. The activated PRP was centrifuged at 1000 × *g* for 6 min, and the supernatant was harvested and stored at 20 °C. The whole blood of 15 mice was collected and used to extract PRP in the same way as described herein. Therefore, the overall concentration of PRP was ~ 3.6 × 10^9^ platelets/mL.

### Compression test

A universal testing machine (ELF3200, Bose, USA) was used to measure the compressive stress–strain of crosslinked GelMA, crosslinked chitosan, and IPN GC hydrogels. The cylindrical hydrogel samples, with height and section diameter of 8 mm, were placed on the measuring platform, and the upper and lower plates (50 mm diameters) were adjusted so they contacted the hydrogel without force. The measurements were recorded until the strain reached 50% and the compression rate was 0.05 mm/s. Three parallel samples were tested for each group. The compressive modulus at 40% stress was defined as the compressive modulus, which was calculated as the slope of the linear portion of the stress–strain curve. Three parallel samples were tested for each sample.

The CellScale Microsquisher (Microtester G2, Waterloo, Canada) was used to measure the IGM compressive stress–strain. One spherical IGM (200 μm) was placed between one plate and a 4 × 4 mm^2^ parallel probe; it was then compressed at a compression rate of 2.5 μm/s. Each IGM was compressed beyond the breaking point. The displacement was continuously tracked and recorded by a camera. That is, when the beam moved to compress the IGM, deflection was measured in real time by the relative position of the beam at the camera and the motor. Meanwhile, the force was continuously calculated and recorded by the Microsquisher software. More specifically, the force–displacement data obtained from the stress were transformed into stress–strain curves. The compressive modulus at 40% stress was defined as the compressive modulus, which was calculated as the slope of the linear portion of the stress–strain curve. Three parallel samples were tested for each sample.

### Swelling test in IGMs

IGMs prepared with a specific concentration of GelMA were defined as weight W_0_ and immersed into a DPBS solution at 37 °C every 10 min for 90 min. The IGMs were then removed from the DPBS solution and wiped dry with filter paper. The swollen weight of IGMs was evaluated as W_t_. The IGM swelling ratio was analyzed using Eq. 2:

swelling ratio (%) = (W_t_− W_0_)/W_0_ (2).

***In vitro*growth factor release studies**.

The release of growth factors in the 10, 15, and 20% GelMA + 1% chitosan microcarriers was analyzed by enzyme-linked immuno sorbent assay (ELISA). After immersion in the PRP solution at 37 °C for 24 h, the prepared IGMs were placed in a 48-well plate (Corning), to which DPBS solution was added. The DPBS was subsequently collected and changed on day 1, 2, 3, 4, 5, 6, and 7. The collected DPBS was placed at − 80 °C. The concentration of FGF, EGF and VEGF were quantified using ELISA kits (Beyotime, Shanghai, China) according to the manufacturer’s instruction.

### Degradation test

The degradation test was performed according to a previously described protocol [70]. The biodegradability was explored via enzymatic degradation experiments and analyzed by weighing the samples at different time points. The samples were weighed as W_0_ immersed in DPBS (pH = 7.4) with 1000 U/mL lysozyme (Aladdin Biochemical Materials Technology) with a stirring rate of 50 rpm at 37 °C for 15 days. At different time points, the samples were removed, washed with DPBS thrice, lyophilized, and weighted as W_t_. The weight loss rate was calculated using Eq. 3:

Weight loss rate = W_t_/W_0_ × 100% (3).

Furthermore, to detect the degradation of 15% GelMA + 1% chitosan microcarriers in vivo, the skin of C57BL/6 mice was injected with IGMs. The mice were sacrificed via cervical dislocation on day 1, 8 and 15 for HE staining analysis. Briefly, the mouse dorsal skin was fixed in 4% paraformaldehyde (Beyotime) at 4 °C, subsequently embedded in paraffin blocks, and prepared as 5-µm-thick sections based on the longitudinal sections of HFs. The samples were then stained with HE, dehydrated, and mounted. Images were obtained with an IX61 FL fluorescence microscope (Olympus, Shibuya-ku, Tokyo).

### Cell cycle assay

Flow cytometry was used to detect the cell cycle of DPCs. After culturing with IGMs loaded with different concentrations of PRP for 36 h, DPCs were harvested and stained with propidium iodide (PI)-RNase (Solarbio). A FACSCanto II flow cytometer (Becton Dickinson, Franklin Lakes, NJ, USA) was used to assess the samples, and the data were analyzed using FlowJo software v.7.6.1.

### CCK-8 assay

A CCK-8 test kit (Meimian, Jiangsu, China) was used to detect the proliferation and toxicity of DPCs treated with IGMs + 0%, 10%, 20% and 30% PRP. After 18 and 36 h, CCK-8 was added to each well, and the plate was incubated for an additional 3 h; the OD value (450 nm wavelength) was measured with a microplate reader (RNE90002, Reagan, Shenzhen, China).

### ***In vitro*** cell migration assay

The influence of different concentrations of the PRP solution loaded into IGMs on cell migration was assessed using a wound-healing scratch assay. DPCs (2 × 10^3^ cells/per well) were added into 24-well plates to form a confluent monolayer. Next, a 20-µL pipette tip (Kejin Biotechnology Co., Ltd, Shanghai, China) was used to scratch a wound within the cell monolayer. After removing the cell debris, DPCs were normally cultured and photographed at 0, 12, 24, and 36 h. The cell migration rate at each point was quantitated using Eq. 4:

Cell migration rate (%) = Ao – At/Ao (4).

where A0 is the scratch area at 0 h, and At represents the scratch area at each time point.

### Western blotting

A RIPA protein lysis buffer (Solarbio) containing a protease inhibitor cocktail was used to isolate protein extracts from DPCs. Next, a BCA protein assay kit (ThermoFisher) was used to measure the absorbance at 562 nm to obtain protein concentrations. Total protein (20 µg) was subjected to SDS-PAGE, and the resolved proteins were transferred to a polyvinylidene fluoride membrane (Millipore, Saint Louis, USA). The membrane was blocked in 5% bovine serum albumin (Solarbio) for 1 h and probed with primary antibodies overnight at 4 °C. The associated primary antibodies were those against alkaline phosphatase (ALP, ab224335, Abcam, Eugene, USA), versican (bs-2533R-A350, Bioss, Beijing, China), neural cell adhesion molecule (NCAM, bsm-52824R, Bioss), and β-catenin (ab32572, Abcam) and β-actin (ab8226, Abcam). After triple washing with Tris-buffered saline Tween (TBST, Solarbio), the membrane was incubated with the corresponding secondary antibody (ab150077, Abcam) for 1 h at 20–25 °C. An Odyssey infrared fluorescence scanning imager (Bio-Rad, Hercules, USA) was used for photography.

### Quantitative reverse transcription-al-time polymerase chain reaction (RT-qPCR)

The total RNA of DPCs was extracted using the TRIzol reagent (ThermoFisher) according to the manufacturer’s instructions. The SYBR Prime-Script RT-PCR Kit (TaKaRa Bio, Gumma, Japan) was used to evaluate the expression of ALP, versican, NCAM, and β-catenin (n = 3 per group). cDNA was amplified under the following conditions: for 30 s at 95 °C, followed by 40 cycles at 95 °C for 30 s, 60 °C for 30 s, and 72 °C for 20 s, using the ABI Prism 7900HT235 sequence detection system (Life Technologies, Carlsbad, USA). The primer sequences for ALP, versican, NCAM, and β-catenin are in Table S1. β-actin was used as the reference gene. Gene expression levels were quantified relative to β-actin levels and calculated using the 2^−∆∆^Ct method.

### Drug treatment of mice after dorsal hair depilation and hematoxylin-eosin of dorsal skin

Seven-week-old male C57BL/6 mice were depilated with hair removal cream to synchronously induce each mouse to initiate the hair cycle. These mice were divided into three groups as follows (n = 4/group): control (direct injection of DPBS), 20% PRP (direct injection of 20% PRP), and 20% PRP-loaded IGMs with specific treatments on days 0, 1, 3, 6 and 9. The skin of mice was evenly injected with 100 µL DPBS or 20% PRP or 20% PRP-loaded IGMs at eight sites to ensure uniform distribution. The speed of the hair cycle transformation from the telogen to anagen phase was analyzed according to the change in skin color and proportion of the novel HF area, as described previously [73, 74]. All mice were examined and photographed on days 0, 5, 9, 13, and 17 and sacrificed using cervical dislocation method on day 5, 9 and 13 for HE staining. The methods of HE staining analysis are described above.

### Isolation and culturing of DPCs from vibrissae follicles of mice

The method of isolating dermal papillae (DP) from the vibrissae follicles of mice was consistent with that described by Xiao et al. [75]. Briefly, the mystacial pad was cut open after cervical dislocation, and fine forceps were used to remove the collagen capsules that surrounded the vibrissae follicles, to expose the follicle bases. Finally, DPs were dissected using thin scissors and placed on the bottom of cell culture dishes after washing thrice with Dulbecco’s modified Eagle’s medium (DMEM, Solarbio) supplemented with 20% fetal bovine serum (FBS, Solarbio), and penicillin-streptomycin-amphotericin B mixed solution (Leagene, Beijing, China). The culture medium was changed every 5 days. After 7–11 days, the DPCs were gathered with 0.25% trypsin-EDTA (Gibco, Brooklyn, USA) and transferred to fresh culture dishes. P4 DPCs were used in this study.

### Cellular biocompatibility and proliferation of DPCs co-cultured with IPN GC hydrogels and IGMs

To assess the biocompatibility of DPCs cocultured with IPN GC hydrogels, IPN GC hydrogels were added to a 24-well plate (Corning), to which p4 DPCs (5 × 10^3^/well) were added along with 3 mL of DMEM supplemented with 10% FBS, penicillin-streptomycin-amphotericin B mixed solution, with or without 20% PRP. The plates were then incubated at 5% CO_2_ and 37 °C. A calcein-AM/PI staining kit (Solarbio) was used to detect biocompatibility of the IPN GC hydrogels (with or without PRP) after incubation for 24 h. On days 1, 4, and 7, the DPCs were stained with propidium iodide (PI, 4 µM) and calcein-AM (2 µM) in DPBS for 15 min at 23 °C after triple washing with DPBS. The stained cells were observed with a confocal laser microscope (LSM800, Zeiss, Jena, Germany). Calcein-AM-stained living cells resulted in green fluorescence, while PI-stained dead cells resulted in red fluorescence. A CCK-8 test was performed as described above.

Similarly, to detect cellular biocompatibility and the proliferation of DPCs cocultured with IGMs and IGMs + 20% PRP, a certain amount of IGMs and IGMs + 20% PRP were added to a 24-well ultra-low attachment culture plate (Corning), to which DPCs (5 × 10^3^/well) were added. Given that the DPCs tend to adhere to the surface of the IGMs and IGMs + 20% PRP, they were cultured with 1.5 mL of DMEM, supplemented with 10% FBS and penicillin-streptomycin-amphotericin B mixed solution, at 5% CO_2_ and 37 °C. A calcein-AM/PI staining kit (Solarbio) was used to detect the biocompatibility of the IGMs on day 1, 4 and 7. A CCK-8 test was performed as described above.

Additionally, based on the above-described method, viability of the DPC aggregates was assessed on day 1, 4, and 7. DPC aggregates were formed by culturing 5 × 10^3^/well DPCs in a 96-well ultra-low attachment plate (Corning) for 8 h at 37 °C. The subsequent staining process was performed according to the above-described protocol.

### Immunofluorescence staining analysis

The samples were triple-washed with DPBS, fixed in 4% paraformaldehyde for 35 min, triple-washed with DPBS, permeated with 0.3% Triton X-100 (Solarbio), and blocked with 3% BSA (Solarbio). They were then stained with primary antibodies against Ki67 (ab15580, 1:200, Abcam), ALP (1:200), versican (1:200), NCAM (1:200), and β-catenin (1:200) for 12 h at 4 °C. Next, the DPCs were stained with Alexa Fluor-488 (ab150077, 1:200, Abcam) and Alexa Fluor-594 (ab150080, 1:200, Abcam) conjugated goat anti-rabbit secondary antibodies for 2 h at approximately 23 °C. The DPC-stained nuclei were counterstained with 2-(4-amidinophenyl)-6-indolecarbamidine dihydrochloride (DAPI, ab285390, 1:200, Abcam). The samples were observed using a confocal laser microscope. Positive areas and fluorescence intensity were analyzed using image Pro-Plus 6.0 software.

### ***In vivo*** fluorescence imaging and hair regeneration

Epidermal cells of newborn mice were prepared as described by Xiao et al. [75]. In brief, the skin of neonatal C57BL/6J mice was mechanically separated and immersed in 0.1% dispase (Solarbio) at -4 °C for 12 h following cervical dislocation. The skin samples were then separated into the epidermis and dermis using forceps. The epidermis was minced with scissors and immersed in 0.025% trypsin (Solarbio) at 37 °C for 20 min. Subsequently, an equal volume of DMEM was added to the samples to terminate the reaction. Then, 40-µm strainers (Solarbio) were used to filter the samples. Murine neonatal epidermal cells were obtained after centrifugation and washing.

DPCs stained with 5 mM green 3,3′-dioctadecyloxacarbocyanine perchlorate (DIO, Beyotime) were seeded on IGMs and 20% PRP-laden IGMs for 7-day cultures. Using laser confocal microscopy, the DPCs on IGMs were enumerated using the Z-stack function of ZEISS Zen Lite 3.5. Next, EPCs were loaded in the GelMA (2% w/v). DPC-laden IGMs with or without 20% PRP were then mixed with EPC-loaded GelMA to form the DECHS and 20% PRP-loaded DECHS groups, respectively. Based on the number of cells in the DECHS and 20% PRP-loaded DECHS groups, the same number of DPCs and EPCs were added to the control mixture. Nine biological replicate sites, containing the same number of cells (2.55 × 10^5^ DPCs and 0.90 × 10^5^ EPCs), were included for each group. A 10 mL syringe with a 21G blunt needle (internal diameter: 600 mm, FeiBo, Xiamen, China) was injected into the hypodermis of nude mice to form a bleb. In addition, 2.55 × 10^5^ DPCs were manually added to 51 wells of 96-well ultra-low attachment culture plates to form 51 aggregates, which were cultured for 7 days, collected and mixed with 0.90 × 10^5^ EPC, and subsequently injected into the mice. In addition, DPC/PRP-loaded IGMs were assessed alone, without combination with EPCs.

DIO-labeled DPCs were detected using an In-Vivo FX Pro imaging system (Bruker, Billerica, Massachusetts, USA) on day 1, 4, 7. The fluorescence intensities of the three groups were measured with the MISE software and expressed as photon flux (photons sec^− 1^, cm^− 2^ sr [SR]^−1^).

After 17 days, the injection area was dissected, and a stereomicroscope (Stemi2000-C, Zeiss) was used to observe and photograph the injection location at various magnifications. The number of novel HFs was quantified using Image Pro-Plus 6.0 software. Samples of the hair regeneration areas were then selected for HE staining as described above.

### Statistical analysis

Experiments were conducted with at least three replicates for each sample (n ≥ 3 per sample). Data were presented as mean ± standard deviation, and significant differences were calculated using Student’s *t* test or a one-way analysis of variance (ANOVA). Values with p < 0.05 were considered statistically significant. Data were quantitated using Image Pro-Plus 6.0 software, GraphPad Prism 9.3.1, Origin 8.5, and SPSS 13.0.

## Electronic supplementary material

Below is the link to the electronic supplementary material.

Supplementary Material 1

Supplementary Material 2

Supplementary Material 3

## Data Availability

All data generated or analyzed during this study are included in this published article and its supplementary information files.

## List of abbreviations

ADC: anchorage-dependent cell.
DECHS: DPC/EPC co-cultured hydrogel system.
DPC: dermal papilla cell.
ECM: extracellular matrix.
EPC: epidermal cell.
FT-IR: Fourier transform infrared.
GAG: glycosaminoglycan.
GC: GelMA/chitosan.
GelMA: gelatin methacryloyl.
HE: hematoxylin-eosin.
HF: hair follicle.
IGM: IPN GelMA/chitosan microcarrier.
IPN: interpenetrating network.
MSC: mesenchymal stem cell.
PDGF: platelet-derived growth factor.
PRP: platelet-rich plasma.
SEM: scanning electron microscopy.
TCP: tissue culture polystyrene.
TGF-FGF: transforming growth factor-fibroblast growth factor
VEGF: vascular endothelial growth factor.
