# Supplementary material for: Scalable and high-throughput production of an injectable platelet-rich plasma (PRP)/cell-laden microcarrier/hydrogel composite system for hair follicle tissue engineering
Source: J Nanobiotechnology. 2022 Nov 3;20:465. doi: 10.1186/s12951-022-01671-8 (PMC9632161; doi:10.1186/s12951-022-01671-8)
Supplement: Supplementary file 1 — Supplementary Material 1 [file 12951_2022_1671_MOESM1_ESM.docx]

**Additional file 1**

**Scalable and high-throughput production of an injectable platelet-rich plasma (PRP)/cell-laden microcarrier/hydrogel composite system for hair follicle tissue engineering**

Yufan Zhang^1‡^, Panjing Yin^2,3‡^, Junfei Huang^1‡^, Lunan Yang^1^, Zhen Liu^1^, Danlan Fu^1^, Zhiqi Hu^1^*, Wenhua Huang^2,3,4^*, Yong Miao^1^*

*
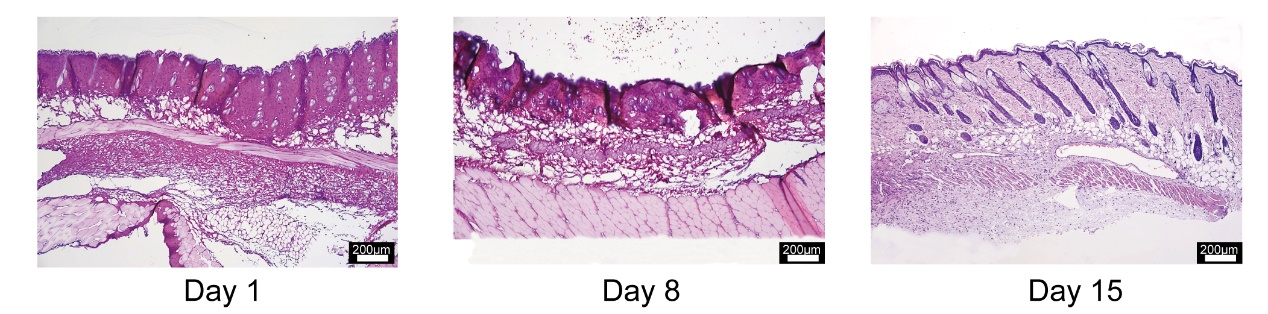
*

Figure S1. HE-stained images showing the degradation of 15% GelMA + 1% chitosan microcarriers *in vivo.*


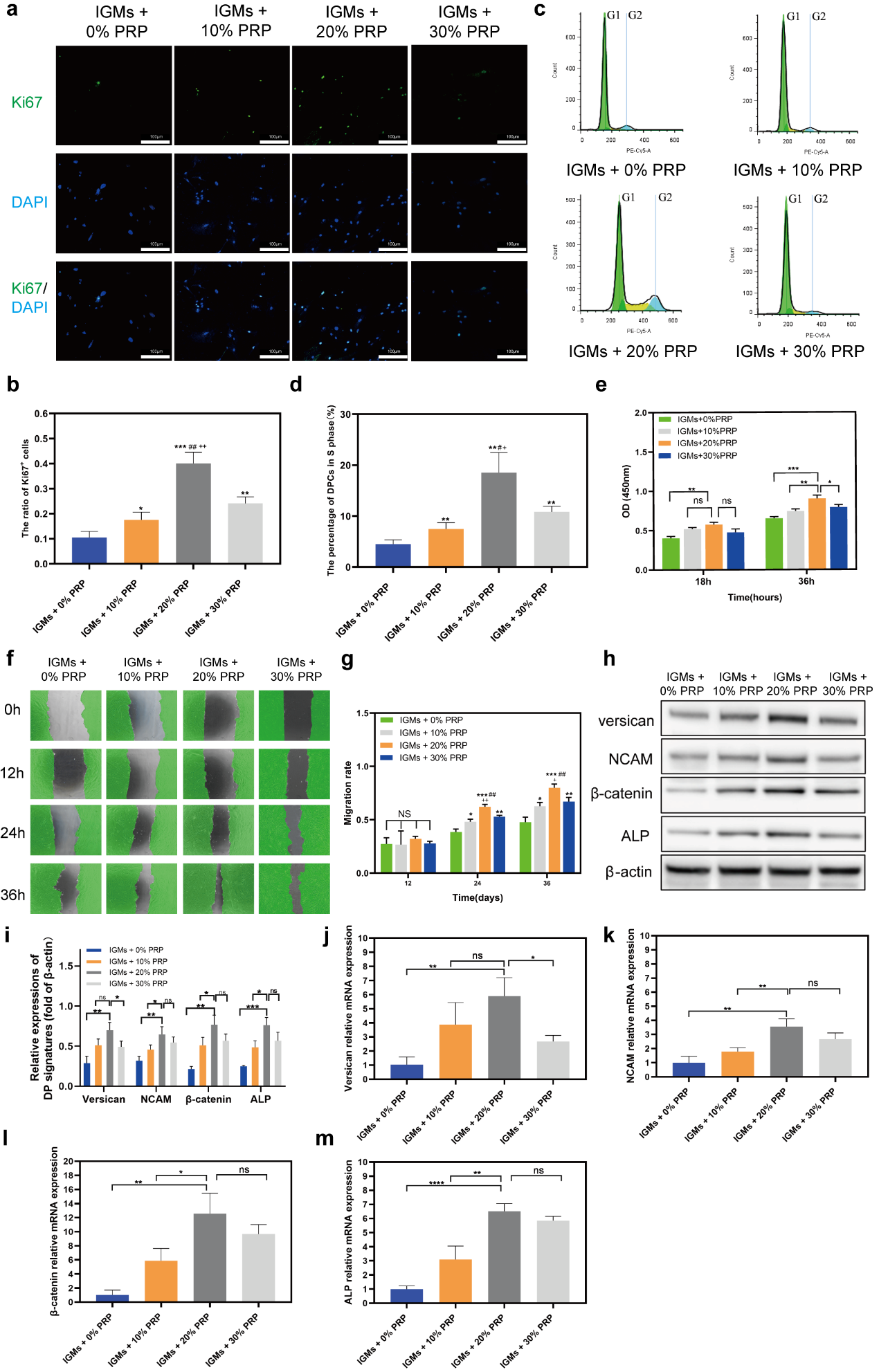


Figure S2. PRP-loaded IGMs promote the proliferation and migration of DPCs. (a) Immunofluorescence images of Ki67 in DPCs. (b) Statistical analysis of the Ki67+ cell proportion in panel a. (c) Flow cytometry profile of the DPC cell cycle. (d) Quantification of DPCs in S phases. (e) Statistical diagram of cell proliferation activity using CCK-8 assay. (f) Microscopy images of DPCs in a wounded area after incubation treatment. (g) Statistical analysis of the migration rate in the wound area from panel f. (h, i) western blotting assays and Q-PCR (j, k, l, m) showing the expression of ALP, versican, NCAM, and β-catenin among the four groups. *p < 0.05 relative to IGMs + 0% PRP; **p < 0.01 relative to IGMs + 0% PRP; ***p < 0.001 relative to IGMs + 0% PRP; #p < 0.05 relative to IGMs + 10% PRP; ##p < 0.01 relative to IGMs + 10% PRP; ++p < 0.01 relative to IGMs + 30% PRP; all values are displayed as means ± S.D.; n = 3 individual experiments.


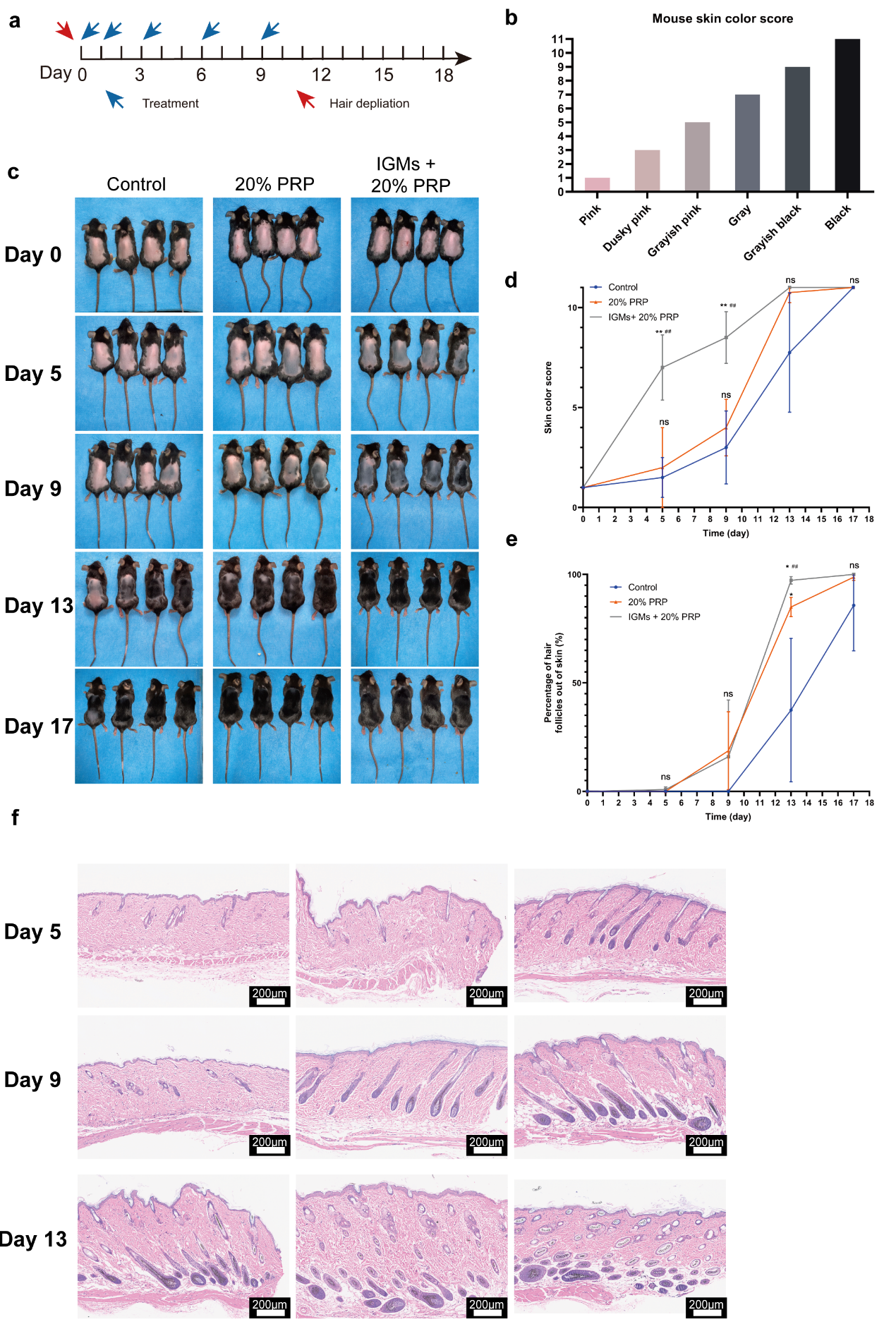


Figure S3. PRP-loaded IGMs enhance PRP therapeutic effects, accelerating the transition from telogen to anagen. (a) Schedule of skin depilation and treatment. (b) Scoring system for mouse skin color. (c) Photographs of dorsal hair regrowth in C57BL/6 mice after treatment. (d) Quantification of skin color score in three groups based on the scoring system in panel b. (e) Evaluation of areas with hair regrowth in panel b. (f) HE-stained sections of dorsal skin with HFs after 5, 9 and 13 days. *p < 0.05 relative to the DPBS group (control); **p < 0.01 relative to DPBS group (control); ***p < 0.001 relative to DPBS group (control); #p < 0.05 relative to 20% PRP group; ##p < 0.01 relative to 20% PRP group; statistical significance was analyzed using unpaired independent-samples t-test; n = 4 for each group.


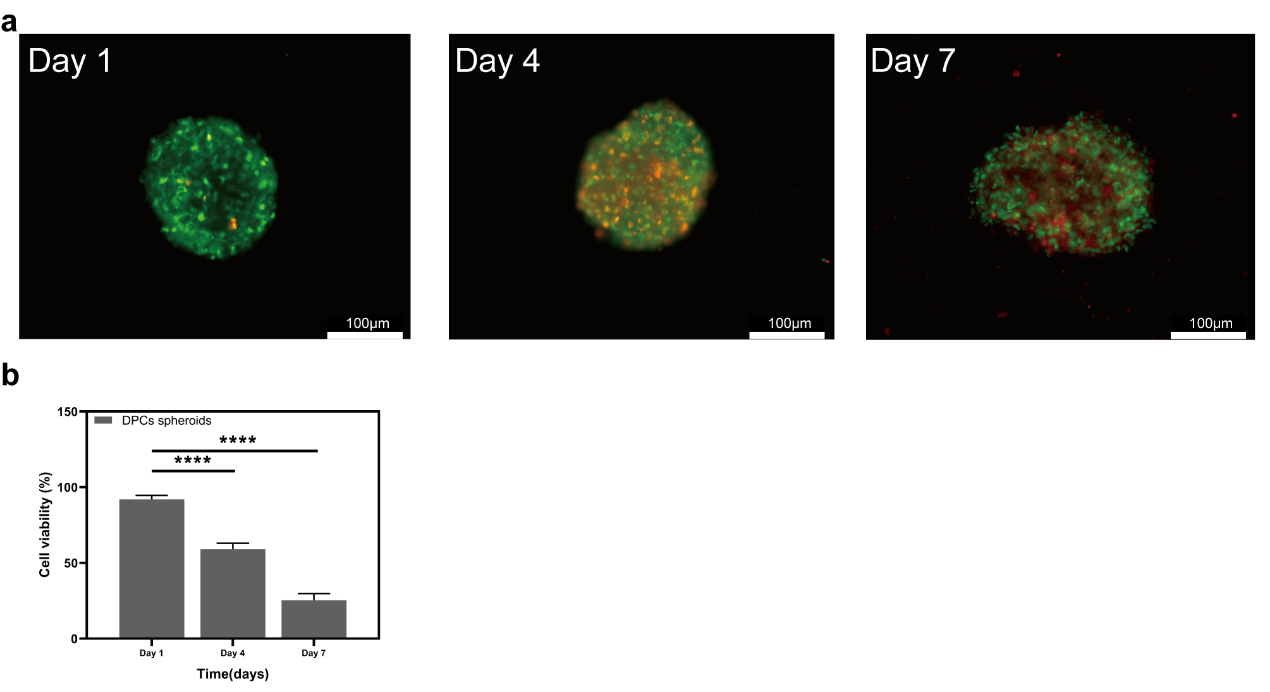


Figure S4. Cell viability of DPC spheroids. (a) DPC spheroids for 1, 4, and 7 days detected by live/dead staining. (b) Statistical diagram of DPC cell viability at day 1, 4 and 7 (****p < 0.0001).


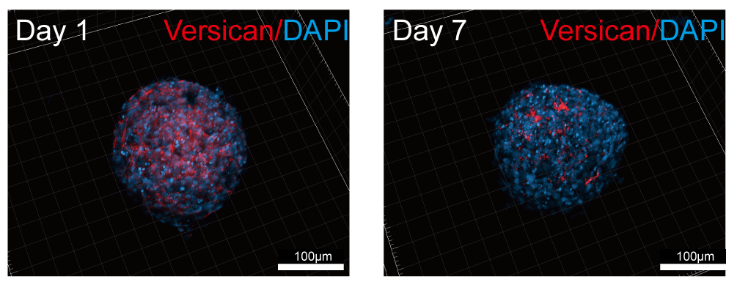


Figure S5. Expression of versican by DPCs spheroids for 1 and 7 days as detected by live/dead staining


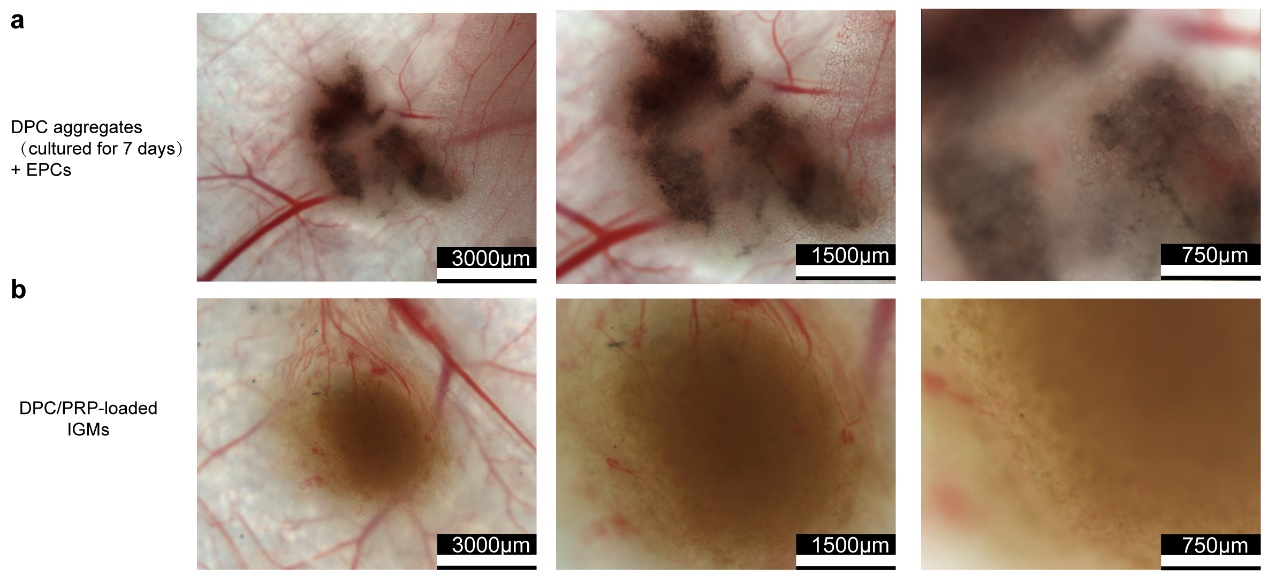


Figure S6. Stereomicroscopic images of injection sites in the DPC spheroid (cultured for 7 days) group (a) and DPC/PRP-loaded IGMs (b).


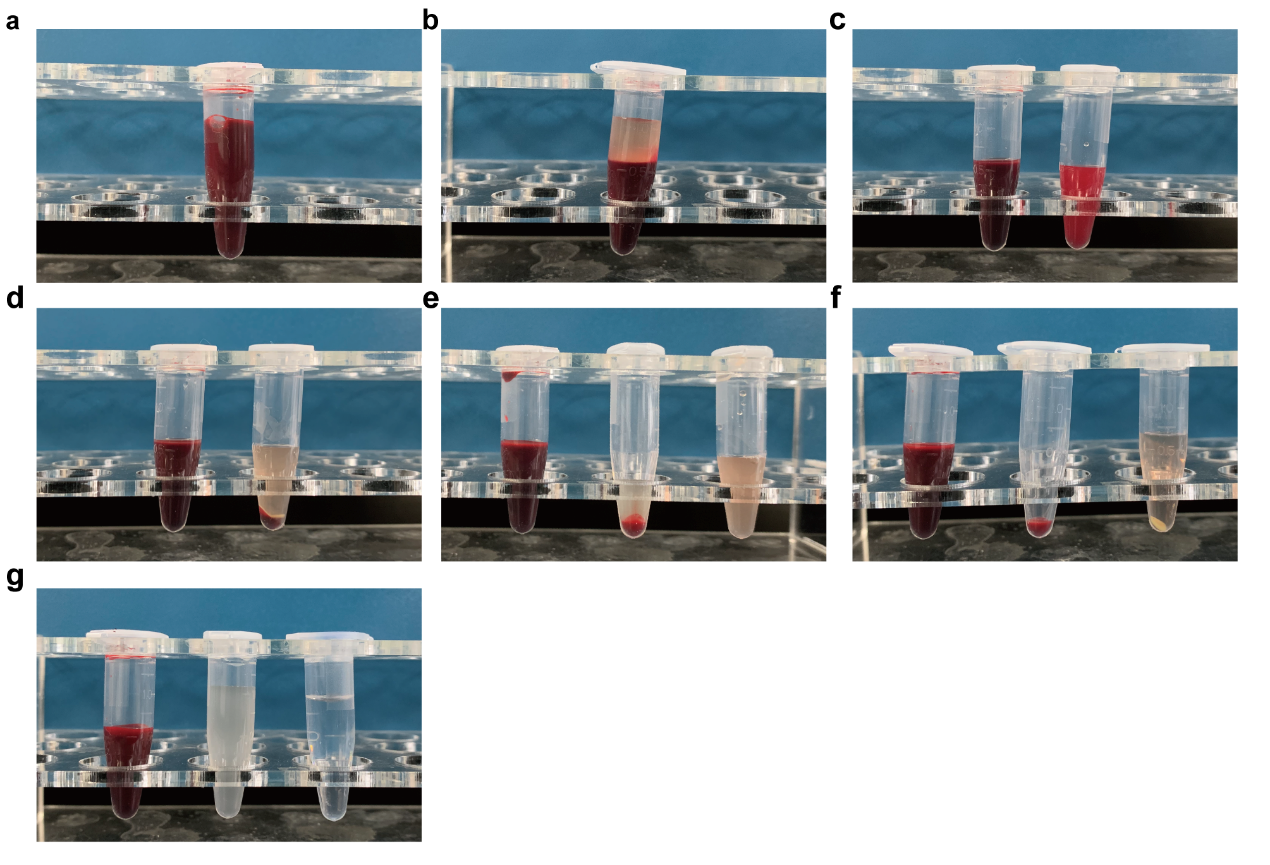


Figure S7. (a-g) PRP extraction process.

**Table S1** Primer sequences for ALP, versican, NCAM, and β-catenin mRNA

| Primer | Sequence (5′-3′) |
| --- | --- |
| *Alp* forward | AACATGTGCCAGACAGTGGA |
| *Alp* reverse | GTTGCACTGGTTAAAGCGGG |
| *Ctnnb1* forward | ATGGCCATGGAACCAGACAG |
| *Ctnnb1* reverse | TGGTAGTGGCACCAGAATGG |
| *Ncam* forward | CTCCACTGCGGACGAATCC |
| *Ncam* reverse | ATTCTTCCTGCGCCTCCCTCTTT |
| *Vcan* forward | TGGTAATGGCTGCATTGGATGA |
| *Vcan* reverse | TGCAAAGCCTGAAAGTAATCAGGG |

*Ctnnb1,* encodes β-catenin; *Vcan* encodes Versican
